# Fragmentation trees reloaded

**DOI:** 10.1186/s13321-016-0116-8

**Published:** 2016-02-01

**Authors:** Sebastian Böcker, Kai Dührkop

**Affiliations:** Friedrich-Schiller-University, Ernst-Abbe-Platz 2, 07743 Jena, Germany

**Keywords:** Mass spectrometry, Metabolites, Natural products, Computational methods, Fragmentation trees

## Abstract

**Background:**

Untargeted metabolomics commonly uses liquid chromatography mass spectrometry to measure abundances of metabolites; subsequent tandem mass spectrometry is used to derive information about individual compounds. One of the bottlenecks in this experimental setup is the interpretation of fragmentation spectra to accurately and efficiently identify compounds. Fragmentation trees have become a powerful tool for the interpretation of tandem mass spectrometry data of small molecules. These trees are determined from the data using combinatorial optimization, and aim at explaining the experimental data via fragmentation cascades. Fragmentation tree computation does not require spectral or structural databases. To obtain biochemically meaningful trees, one needs an elaborate optimization function (scoring).

**Results:**

We present a new scoring for computing fragmentation trees, transforming the combinatorial optimization into a Maximum A Posteriori estimator. We demonstrate the superiority of the new scoring for two tasks: both for the de novo identification of molecular formulas of unknown compounds, and for searching a database for structurally similar compounds, our method SIRIUS 3, performs significantly better than the previous version of our method, as well as other methods for this task.

**Conclusion:**

SIRIUS 3 can be a part of an untargeted metabolomics workflow, allowing researchers to investigate unknowns using automated computational methods.

**Graphical abstract:**
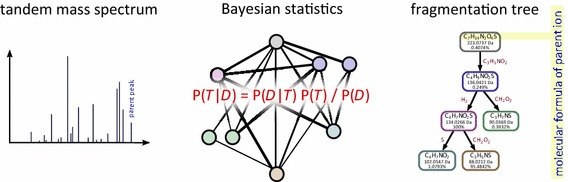
We present a new scoring for computing fragmentation trees from tandem mass spectrometry data based on Bayesian statistics. The best scoring fragmentation tree most likely explains the molecular formula of the measured parent ion

## Background

Liquid chromatography mass spectrometry (LC–MS) is one of the predominant experimental platforms for untargeted metabolomics. With advances in mass spectrometry instrumentation, it is possible to detect 1000s of metabolites simultaneously from a biological sample [1, 2]. Untargeted metabolomics comprehensively compares the intensities of metabolite peaks between two or more samples. Here, a major challenge is to determine the identities of those peaks that exhibit some fold change [2], a central task in chemical analysis [3]. Tandem mass spectrometry (MS/MS) using collision-induced dissociation (CID) fragments molecules into smaller parts; fragmentation spectra can be used to examine the metabolite’s structure and, ultimately, to elucidate its identity. A significant bottleneck is the interpretation of the resulting tandem mass spectra.

Tandem mass spectrometry data is usually searched against spectral libraries [3–6]. Computational methods exist that target compounds not contained in a spectral library [7, 8]: in particular, several methods try to replace spectral libraries by more comprehensive molecular structure databases, for searching [9–19]. But these methods fail for those compounds not present in a structure database.

**Fig. 1:**
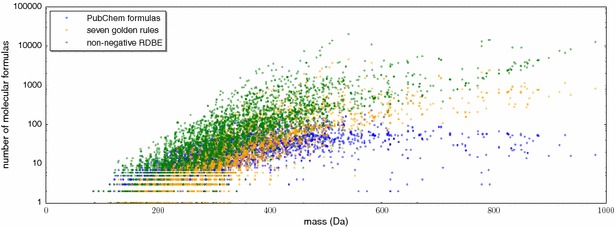
Number of molecular formulas that match the mass of some precursor peak in the Agilent and GNPS dataset, using the maximum of 10 ppm and 2 mDa as allowed mass deviation. Note the logarithmic scale of the y-axis. SIRIUS 3 restricts the set of candidate molecular formulas solely by the non-negative ring double bond equivalent (RDBE) rule (*green*), see (3). More restrictive filtering such as the Seven Golden Rules [20] (*orange*) further reduce the number of molecular formulas to be considered; nevertheless, multiple explanations remain for most precursor ions. We find that 1.6 % of the compounds in our datasets *violate* the Seven Golden Rules. We also report the number of molecular formulas found in PubChem for the above mentioned mass accuracy

Identifying the molecular formula of a compound is already a challenging problem: most peaks in an LC–MS run are ambiguous and can be explained by several molecular formulas, even when using instruments with high mass accuracy. This is particularly the case for compounds above 400 Da (see Fig. 1). Molecular formula constraints [20] reduce the diversity of possible explanations but by themselves cannot solve the underlying problem. It is understood that by applying more restrictive filters, we may filter out the correct molecular formula, limiting novel discoveries; this is particularly the case if we restrict ourselves to molecular formulas from some molecular structure database such as PubChem. Methods for predicting the molecular formula of an unknown compound usually require data beyond tandem mass spectra [21–23]. In particular, several methods successfully use isotope patterns for this purpose [20, 24–29]: as an example, the SIRIUS isotope pattern analysis [27] was able to correctly identify the molecular formula of 10 out of 13 compounds of the Critical Assessment of Small Molecule Identification (CASMI) contest (http://www.casmi-contest.org/) 2013, without using any fragmentation pattern information [30]. In contrast, network-based methods [31–33] usually do not aim at the identification of a single molecular formula or compound.

**Fig. 2:**
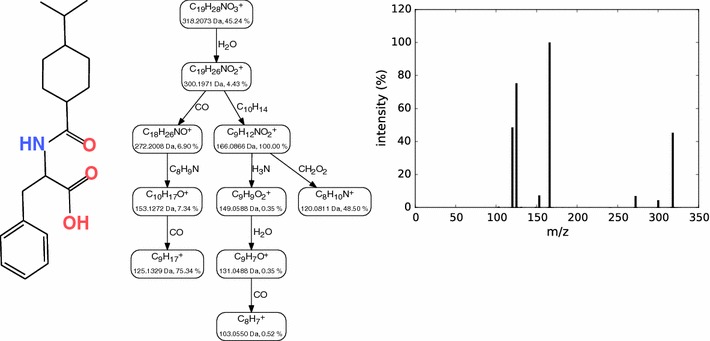
Example of a fragmentation tree. *Left* the molecular structure of Nateglinide. *Right* the measured MS/MS spectrum of Nateglinide from the GNPS dataset. *Middle* the FT computed from the MS/MS spectrum. Each node is labeled with the molecular formula of the corresponding ion, and each edge is labeled with the molecular formula of the corresponding loss. For nodes, we also report m/z and relative intensity of the corresponding peak. We stress that the FT is computed without any knowledge of the molecular structure and without using any database, but solely from the MS/MS spectrum

Fragmentation trees (FTs) were introduced by Böcker and Rasche [34]. FTs annotate the MS/MS spectra and also model the fragmentation processes that generated the fragment ions: each node in the FT assigns a molecular formula to a fragment peak, whereas edges represent fragmentation reactions and are labeled with the molecular formula of the corresponding loss. Peaks for which no node exists in the tree are considered noise. The molecular formula of the FT root is the putative molecular formula of the precursor ion. See Fig. 2 for an example of a FT.

Clearly, the term “fragmentation tree” has been used much earlier than 2008 in the MS literature [35, 36]; the important difference is that FTs in [34] are computed directly from the data by an automated method, without knowing the molecular structure of the compound, and *without the need for a database* of tandem mass spectra or molecular structures. We stress that FTs are computed using tandem MS data; one can also do so using multiple MS data [37, 38] but this is not a requirement. This is fundamentally different from “spectral trees” [39] which solely describe the experimental setup of a multiple MS experiment; see the review by Vaniya and Fiehn [40] on the subject.

FTs can be used to identify the molecular formula of a compound [34]: for each molecular formula decomposition that is within the given mass accuracy of the parent peak, find the FT rooted with this molecular formula that has maximum score. Then, rank the FTs and, hence, the molecular formulas according to the obtained scores. The scoring used in [34] was further evolved in [41, 42], in particular by including a list of radical losses which are not considered implausible, compare to Table 2.

Rasche et al. [41] also showed that FTs can contain viable structural information about an unknown compound. In particular, computed FTs were manually evaluated by MS experts. For 79 FTs having a total of 808 losses, they found that more than 78 % of the losses were annotated as “correct” by MS experts. Rasche et al. [42] showed that FT *alignments* can be used to derive information about a compound’s molecular structure, beyond the molecular formula of the compound: in particular, FT alignments can be used to search a spectral library for a compound which is structurally similar (but not identical) to the query compound, in case the query compound itself is missing from the database [43, 44].

The computational problem underlying FT computation has been coined the Maximum Colorful Subtree problem [34]; unfortunately, this problem is computationally hard [45]. Nevertheless, there exist a number of algorithms (both exact and heuristic) to solve the problem in practice [34, 45, 46]. Here, we will not cover any algorithmic details of the problem; we solve our instances exactly using integer linear programming (ILP) as described in [45]. Compared to the original Dynamic Programming algorithm from [34], the ILP is several orders of magnitude faster, allowing us to consider more peaks in the computation.

In this paper, we report a systematic approach for choosing the fragmentation tree that best explains the observed data, based on Bayesian analysis and a Maximum A Posteriori estimation. Our Maximum A Posteriori estimate roughly follows the scorings from [34, 41, 42]. In contrast to theirs, our approach does not rely on an expert-curated list of common losses; instead, common losses and their frequencies are learned from the data. To calculate the posterior probability of a FT, we propose models to estimate its prior probability and its likelihood. The prior probability is independent of the experimental MS/MS data, whereas the likelihood is the probability of the data, given the model. We estimate hyperparameters for determining the prior probability using experimental data; these hyperparameters are part of the released software and do not have to be retrained for applying the method. In contrast, parameters for mass accuracy and peak intensities used for estimating the likelihood of a FT can be set individually for every analysis. Finally, our method SIRIUS 3 uses hypothesis-driven recalibration from [47].

We evaluate FTs using two derived measures: both for the identification of molecular formulas of unknown compounds, and for searching a database for chemically similar compounds, the new FTs perform significantly better than state-of-the-art methods. In particular, SIRIUS 3 performs significantly better than its predecessors for molecular formula identification. We argue that this is due to an increase in quality of the FTs computed by SIRIUS 3. We stress that SIRIUS 3 is *not* restricted to molecular formulas from any database. Evaluation is carried out on data from several 1000 compounds and two independent datasets.

The implementation of the method presented here is freely available from our website (http://bio.informatik.uni-jena.de/software/) as version 3.0 of the SIRIUS framework for MS and MS/MS analysis.

## Results and discussion

**Fig. 3:**
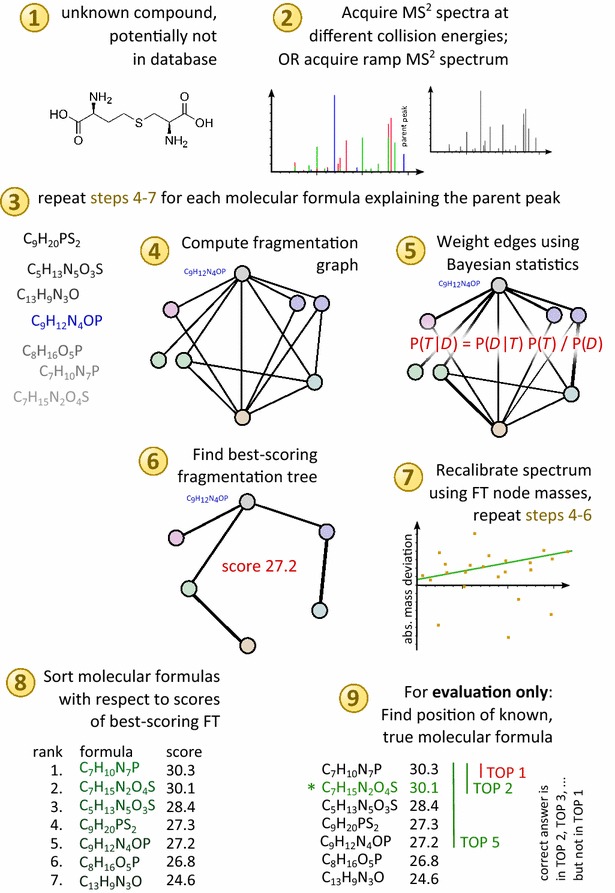
Analysis Workflow. After importing the tandem mass spectra of a compound, all molecular formulas within the mass accuracy of the parent peak are generated (*3*). Each of these candidates is then scored (*4*–*7*) and, finally, candidates are sorted with respect to this score (*8*). To score a candidate molecular formula, we compute the fragmentation graph with the candidate formula being the root (*4*); score the edges of the graph using Bayesian statistics (*5*); find the best-scoring FT in this graph using combinatorial optimization (*6*); finally, we use hypothesis-driven recalibration to find a best match between theoretical and observed peak masses (*7*), recalibrate, and repeat steps (*4*–*6*) for this candidate formula. In our evaluation, we compare the output list with the true answer (*9*)

See Fig. 3 for a schematic workflow of SIRIUS 3.

Evaluating the quality of the FTs computed by SIRIUS 3, is a non-trivial problem. There is practically no way to determine the *ground truth* of the fragmentation process: even the comparison with fragmentation cascades obtained using MS^*n*^ data is not a fully satisfactory solution, as fragmentation at high energy using MS/MS can differ substantially from the multi-step fragmentation with low energy in an MS^*n*^ instrument. Recall that expert evaluation of FTs in [41] resulted in 78 % of all losses being annotated as “correct”; this may serve as an indication of the overall quality of fragmentation trees. Unfortunately, manual evaluation is very work-intensive and, hence, infeasible for the two large-scale datasets considered here; furthermore, it only tests whether our computations are in agreement or disagreement with what MS experts believe, but not necessarily the ground truth.

Since we cannot directly evaluate the quality of our hypothetical FTs, we resort to a different method: we evaluate the performance of SIRIUS 3 in answering a question where the true answer is known. The idea behind our evaluation is as follows: if SIRIUS 3 is capable of identifying the correct molecular formula of a compound, then this is an indication that the structure of the FT is at least somewhat meaningful. More importantly, if results *improve* through subtle modifications of the scoring, then this is an indication that FT quality did also improve.

### Molecular formula identification

We identify the molecular formula of a compound as proposed in [34]: for each molecular formula decomposition that is within the given mass accuracy of the parent peak, find the FT rooted at this molecular formula with maximum score (maximum posterior probability). Then, rank the FTs and, hence, the molecular formulas according to the reached posterior probability. As the true molecular formula is known for all compounds in our datasets, we can then evaluate the rank of the true molecular formula in the output.

For evaluation we use two MS/MS datasets called “Agilent” (2046 compounds) and “GNPS” (2005 compounds), see “Experimental” section for details. Compounds in the two datasets are composed from chemical elements C, H, N, O, P, S, plus halogens F, I, Cl, and Br, but no other elements. We note that SIRIUS 3 is not restricted to these elements.

We group compounds composed solely from C, H, N, O, P and S into the “CHNOPS” batch, and compounds containing halogen elements into the “contains FClBrI” batch. For batch CHNOPS, SIRIUS 3 is run using this alphabet of elements without any further restrictions. For batch “contains FClBrI” we assume that we know upfront which of the elements, besides CHNOPS, *may* be contained in the compound: for example, for a compound with molecular formula $\hbox {C}_{18} \hbox {H}_{13} \hbox {ClFN}_{3}$ we start our analysis over the alphabet CHNOPSClF, but SIRIUS 3 may still (wrongly) decide that the compound contains no chlorine or fluorine. This covers the case where we have some indications for the presence of these elements, but have to consider false positives. We do not restrict the number of atoms for each elements.

The above evaluation setup implicitly assumes that we can determine if an element is putatively contained in an unknown compound, *before* computing the FT. This classification may be based on the exceptional isotope pattern of compounds containing chlorine and bromine, or the presence of certain losses in the fragmentation spectra for iodine. We argue that doing so is possible for ClBrI with high precision and recall; in fact, SIRIUS 3.0 offers an option to auto-detect chlorine and bromine by simple rules. But fluorine may pose a problem, as it has only a single stable isotope, and may be undetectable using characteristic losses (mass differences) in the MS/MS spectrum. To this end, simulation results may be too optimistic for the 344 compounds containing fluorine.

We evaluate the performance of SIRIUS 3 against existing methods for determining the molecular formula using MS/MS data. As a baseline method to evaluate against, we use the *naïve method* that returns the molecular formula with the smallest mass difference to the measured parent mass. This method completely ignores all fragmentation data, but will nevertheless in some cases find the correct answer, in particular if there are only few possible explanations of the parent mass. This strategy identifies the correct molecular formula for 14.6 % of the instances, and in 31.3 % the correct formula can be found in the top 5. Both of our datasets have no systematic mass error, see Fig. 11 for the GNPS dataset; for datasets that show a systematic mass error, we expect worse identification rates for the naïve method.

Another common approach is to search the neutral parent mass in a compound database. If we restrict our search to molecular formulas that are contained in PubChem, and again rank molecular formula candidates by mass difference to the measured parent mass, we find the correct molecular formula for 17.1 % of the instances in top rank, and 59.8 % in the top 5. This approach is, by design, restricted to molecular formulas that are already known, and must naturally miss cases where no molecular formula is contained in a structure database. The improved performance is, hence, solely based on the reduced number of candidate molecular formulas, in particular for larger masses. We stress again that SIRIUS 3 is *not* restricted to molecular formulas from any database.

Second, we compare SIRIUS 3 against its predecessor, namely the computational method from [41] with the score modifications from [42]. This method has been released as “SIRIUS^2^ (version 1.0)”, and will be referred to here as “SIRIUS^2^-DP”. SIRIUS^2^ does not use the Integer Linear Program proposed in [45] for computing FTs but instead, combines dynamic programming (DP) with a heuristic. This combination of algorithms is possibly inferior to the ILP from [45] used here, so we also combined the old SIRIUS^2^ scoring with the ILP from [45]; this method is referred to as “SIRIUS^2^-ILP” in the following.

**Fig. 4:**
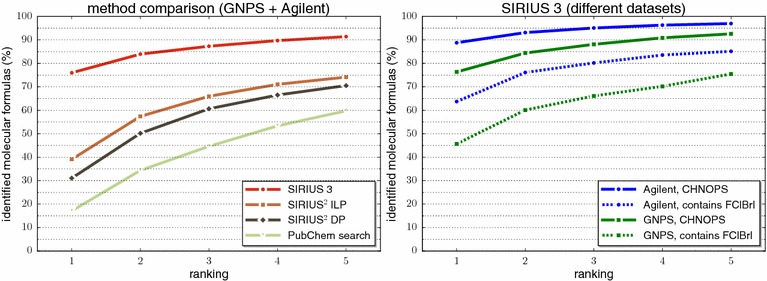
Performance evaluation, percentage of instances (y-axis) where the correct molecular formula is present in the top *k* for $k=1,\ldots ,5$ (x-axis). *Left* performance evaluation for different methods on both datasets. Methods are “SIRIUS 3” (the method presented here), “SIRIUS^2^-ILP” (scores from [41, 42] solved by integer linear programming), “SIRIUS^2^-DP” (scores from [41, 42] solved by dynamic programming), and “PubChem search” (searching PubChem for the closest precursor mass). *Right* performance of SIRIUS 3 for the two compound batches (CHNOPS as solid line, “contains FClBrI” as *dashed line*) and the two datasets (GNPS *green*, Agilent *blue*)

In Fig. 4 we report whether the true molecular formula is contained in the top *k* output of the different methods, for varying *k*. We find that SIRIUS 3 can correctly identify the molecular formula for 76.0 % of the instances, compared to 31.1 % for SIRIUS^2^-DP and 39.1 % for SIRIUS^2^-ILP. Using an ILP [45] instead of the original dynamic programming algorithm does result in both better identification rates and decreased running times. But the better part of performance improvements must be attributed to the new scoring presented here: we observe a 2.5-fold increase of correct identifications when compared to SIRIUS^2^-DP, and roughly a twofold increase when compared to SIRIUS^2^-ILP.

**Fig. 5:**
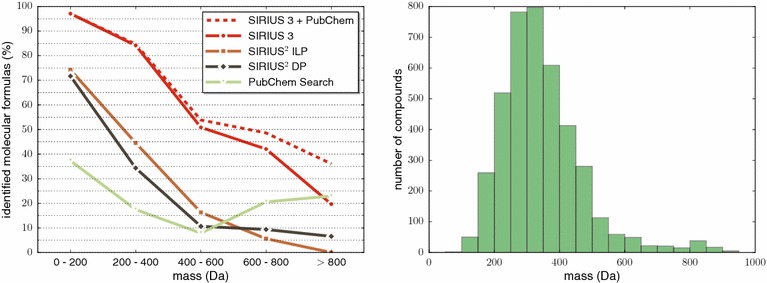
*Left* identification rates of all methods in dependence on the mass of the compound, compare to Fig. 4. Restricting SIRIUS 3 to molecular formulas from PubChem is included for comparison. *Right* histogram for masses of all compounds in the two datasets, bin width 50 Da

Figure 5 shows identification rates as a function of compound mass. Identification rates of SIRIUS 3, SIRIUS^2^-ILP and SIRIUS^2^-DP decrease with increasing mass: this can be attributed to the fact that more candidate molecular formulas have to be considered for larger masses, compare to Fig. 1. Searching the precursor peak mass in PubChem, we observe better identification results for mass bins 600–800 Da and 800+ Da than for mass bin 400–600 Da. As mentioned above, this can be interpreted as an artifact of the distribution of molecular formulas in PubChem: as seen in Fig. 1, the number of candidate molecular formulas in PubChem reaches its maximum for mass bin 400–600 Da. Regarding the distribution of compound masses in the two datasets, we observe that the vast majority have mass below 650 Da, see again Fig. 5.

To support our claim that the observed performance gain when searching large compounds in PubChem is simply an artifact, we also restricted SIRIUS 3 to molecular formulas from PubChem (Fig. 5). We stress once more that unless explicitly stated, SIRIUS 3 will consider *all* molecular formulas. Up to 600 Da, identification rates are on par with SIRIUS 3 considering all possible molecular formulas. For larger mass, identification rates for the smaller PubChem candidate lists outperform the regular SIRIUS 3.

**Fig. 6:**
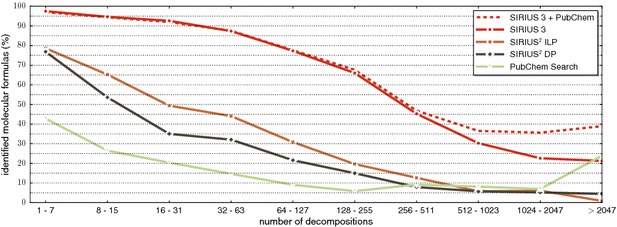
Identification rates of SIRIUS 3, SIRIUS^2^-ILP and SIRIUS^2^-DP depending on the number of candidate molecular formulas: that is, the number of decompositions of the precursor mass that have non-negative RDBE, see (3). Searching PubChem by precursor mass, and restricting SIRIUS 3 to molecular formulas from PubChem are included for comparison

To further elaborate on this point, we show identification rates of SIRIUS 3, SIRIUS^2^-ILP, and SIRIUS^2^-DP as a function of the *number of candidates* in Fig. 6. We observe that indeed, the identification rates of all three methods almost monotonically decrease with increasing number of candidates. Again, SIRIUS 3 shows much better performance than its predecessors: for up to 255 candidates, identification rates of SIRIUS 3 are better than those of SIRIUS^2^-ILP and SIRIUS^2^-DP for 8+ candidates. We have included “searching PubChem for the precursor mass” for comparison; clearly, the number of decompositions is *not* the number of candidates considered by this method. The improved performance for 2048+ decompositions is due to these compounds having large mass and, hence, fewer candidates in PubChem. Again, we also show identification rates for SIRIUS 3 considering only molecular formulas from PubChem.

To the best of our knowledge, the only other method that we could evaluate against, is MOLGEN-MS/MS [48] introduced in 2011 by MOLGEN (Bayreuth, Germany). Here, the fragments are inserted directly below the parent ion, but peak intensities as well as mass deviations of the fragments are taken into account in the scoring. Evaluations by Stravs et al. [49] indicated that MOLGEN-MS/MS is roughly on par with SIRIUS^2^-DP. MOLGEN-MS/MS is commercial, so we cannot estimate its performance on the datasets used here; but we evaluate SIRIUS 3 against MOLGEN-MS/MS on an independent dataset.

#### Adding isotope information

Even better results for identifying molecular formulas can be reached if one combines fragmentation patterns and isotope patterns of an unknown compound. Unfortunately, no isotope pattern information is available for the GNPS and Agilent dataset. To this end, we have to *simulate* the isotope pattern of each compound. Since there is no generally accepted way of how to disturb this ideal data, we use a simple approach suggested repeatedly in the literature, see for example [26]: we ignore peak masses and use peak intensities only. We compare the (undisturbed) simulated isotope distribution of the true molecular formula with the simulated isotope distribution of each candidate molecular formula, by summing the absolute errors of intensities over all peaks. We then filter the best-scoring 20 % (10, 5 %) of all candidate molecular formulas. We do not use the isotope comparison scores in the further analysis: keeping these scores would give an “unfair” advantage for the true molecular formula, as we did not disturb its isotope pattern, and may result in overestimating the method’s power.

**Fig. 7:**
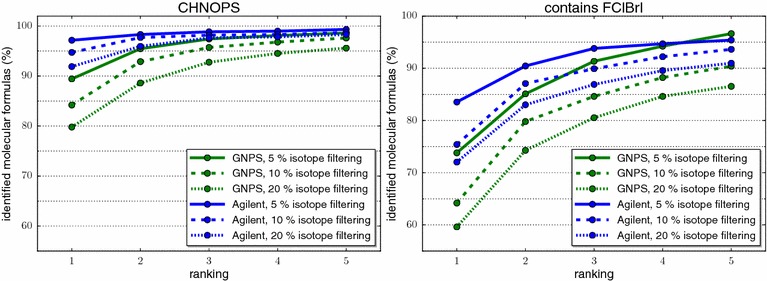
Performance evaluation of SIRIUS 3 when adding isotope information, percentage of instances (y-axis) where the correct answer is present in the top *k* for $k=1,\ldots ,10$ (x-axis). Isotope pattern filtering efficiency 5 % (*solid*), 10 % (*dashed*), and 20 % (*dotted*). Batch CHNOPS (*left*) and “contains FClBrI” (*right*), datasets GNPS (*green*) and Agilent (*blue*)

We report results in Fig. 7. We reach identification rates of 85.6 % for 20 % filtering (89.3 % for 10 % filtering, 93.2 % for 5 % filtering) using the CHNOPS batch, and identification rates 66.8% (70.6, 79.4 %) for the “contains FClBrI” batch with 20 % (10 and 5 %, respectively) filtering. Overall, SIRIUS 3 correctly identifies 81.1 % (84.8, 89.9 %) of the molecular formulas, 89.0 % (92.5, 94.7 %) are in the top 2, and 95.0 % (96.7, 98.2 %) are in the top 5, for 20 % (10, 5 %, respectively) filtering.

#### Running time

**Fig. 8:**
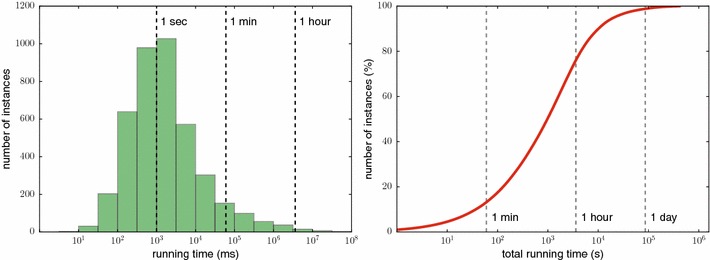
*Left* histogram of running times of all instances (compounds) in the two datasets. *Right* cumulative distribution of running times

We solve all instances of the Maximum Colorful Subtree problem (one instance corresponds to one compound) on a 2× Intel XEON 6 core E5-2630 at 2.30 GHz with 128 GB RAM. We compute 12 instances in parallel, so that only one CPU core is used per process. Running times are given per core, unless indicated otherwise. We solve Integer Linear Programs using Gurobi 6.0 (http://www.gurobi.com/). Processing all compounds from the two datasets requires 114 h of computing time (<1 day of wall-clock time). We find that 45 % of the instances are solved in less than a second, and 93.5 % of the instances require less than a minute. Computing the “easiest” 90 % of all instances requires only 2.8 h; in contrast, the 1 % “hardest” instances use more than 70 % of the total computing time. See Fig. 8 for the distribution of running times.

We stress that computing a single fragmentation tree rarely takes more than a few seconds. But for some instances, we have to compute up to 20,000 FT to consider all possible molecular formulas. For example, the longest-running instance runs for 12.5 h and computes 3106 FTs; hence, each tree is computed within 15 s.

Computation time is not a concern of this paper, as gathering the data required far more time than computing the FTs. There are numerous ways to speed up computations: first and foremost, White et al. [46] have recently presented data reduction techniques and a stronger ILP formulation for the problem, which results in a ninefold decrease of running time for computationally “hard” instances. Second, computation time highly depends on the number of molecular formulas that have to be considered. Using isotope pattern information to upfront select only the, say, 10 % most likely molecular formulas would speed up computations roughly tenfold. Third, if computing time is a major concern, then database search or stricter constraints (such as the Seven Golden Rules [20]) can be used to further limit the number of molecular formulas. As mentioned above, this comes at the risk of excluding the true molecular formula.

#### Evaluation on independent data

To show that we have not “overtrained” SIRIUS 3, we evaluate its performance on two independent datasets. We *do not re-estimate* any hyperparameters for these evaluations but rather use those described above. Only mass accuracy and the set of elements are chosen appropriately.

First, a preliminary version of SIRIUS 3 was used in the CASMI contest 2013 to determine the molecular formula of 12 unknown compounds. Using only the fragmentation tree analysis described here and ignoring the isotope pattern data, we correctly identified eight molecular formulas, and placed an additional three in the top 2 [30]. In conjunction with isotope pattern analysis, we identified 10 out of 12 molecular formulas, and SIRIUS was selected “best automated tool” of the molecular formula challenge [50].

Second, we evaluate SIRIUS 3 on independent data by Stravs et al. [49]. This dataset contains 60 compounds (pesticides) with mass between 191.1 Da (DEET) and 443.1 Da (Propaquizafop). Here, 28 compounds contain a halogen element. Both isotope patterns and MS/MS data are provided. We use the alphabet of elements CHNOPSClBrI for all instances, and mass accuracies 5 and 10 ppm as suggested in [49]. Results of MOLGEN-MS/MS and SIRIUS^2^-DP are taken from [49].^1^ For evaluation purposes, we estimate the power of SIRIUS 3 using only MS/MS data, and we also combine results of isotope pattern and MS/MS analysis as described in [30]. See Table 1 for details. We find that combining MS/MS and isotope pattern data, SIRIUS 3 correctly identifies molecular formulas for 93.3 % of the instances and for 26 of 28 halogenated compounds, clearly outperforming both MOLGEN-MS/MS and SIRIUS^2^-DP. When omitting the isotope pattern data, SIRIUS 3 still identifies the correct molecular formula for 81.6 % of the instances, and for 18 out of 28 halogenated compounds.

**Table 1:** Performance comparison of SIRIUS 3 with MOLGEN-MS/MS using 60 compounds from [49], uncalibrated spectra

	MOLGEN-MS/MS		SIRIUS^2^-DP		SIRIUS 3			
	With isotopes		With isotopes		Without isotopes		With isotopes	
	10 ppm	5 ppm	10 ppm	5 ppm	10 ppm	5 ppm	10 ppm	5 ppm
Top 1	36	34	34	35	49	45	55	56
Top 2	44	47	50	46	51	51	58	58
Top 5	54	55	52	48	58	*60*	60	60
Average rank	2.55	2.30	1.57	1.63	1.58	1.5	1.17	1.15
Worst rank	23	20	11	15	10	*5*	5	5

All tools are run with mass accuracy parameter 5 and 10 ppm. Best entries in italics. Results for MOLGEN-MS/MS and SIRIUS^2^-DP taken from [49]. In that evaluation, SIRIUS^2^-DP crashed 7/5 times for 10/5 ppm mass accuracy, and did not consider the correct molecular formula of the compound for 0/6 compounds

Third, we use MS/MS spectra of 874 compounds from MassBank as independent data, see “Experimental” section for details. Masses in this dataset range from 82.1 to 901.2 Da; 54 compounds contain halogens. The “MassBank” datasets consists of eight sub-datasets measured on QTOF and Orbitrap instruments. In total, SIRIUS 3 correctly identifies molecular formulas of 668 compounds (76.4 %). It reaches best identification rates for the FIOCRUZ and UFZ sub-dataset (more than 94 %), and worst identification rate for the NAIST sub-dataset (54 %).

#### Chemical prior evaluation

In “Prior probability of the tree” section we define chemical priors that estimate whether a molecular formula candidate is “reasonable” for any metabolite. Such prior knowledge about “reasonable” molecular formulas has been discussed repeatedly in the literature, most notably by Kind and Fiehn [20]. It is important to understand that for SIRIUS 3, these priors are no filters: that is, molecular formulas which violate any of the prior assumptions, are *not discarded* but only penalized, as they are assumed to be less probable. Furthermore, prior probabilities are chosen conservatively, so that “unlikely” molecular formulas are penalized only slightly: if there is sufficient MS/MS data, SIRIUS 3 will “overrule” these priors, returning a molecular formula that violates one or even several prior assumptions. For example, we find that SIRIUS 3 *correctly identifies* the following molecular formulas, although the “hetero minus oxygen to carbon ratio” prior is violated: $\hbox {C}_{8} \hbox {H}_{15} \hbox {N}_{7} \hbox {O}_{2} \hbox {S}_{3}$ (famotidine), $\hbox {C}_{6}\hbox {H}_{8} \hbox {ClN}_{7}\hbox {O}$ (amiloride), and $\hbox {C}_{2} \hbox {H}_{8} \hbox {NO}_{2} \hbox {PS}$ (methamidophos). Note that the third molecular formula is additionally penalized by the RDBE prior, as it has ring double bond equivalent of zero. We find that for about 40 compounds in the GNPS and Agilent datasets, the correct molecular formula receives a considerable prior penalty; out of these, SIRIUS 3 identifies the correct molecular formula for 25 compounds (62.5 %).

Second, although hyperparameters of the priors are determined from molecular structure databases, we do not train our method using these databases. Rather, we assume that the prior assumptions will hold for *any biomolecule*, and use the molecular structure database solely to estimate the hyperparameters.

Third, SIRIUS 3 can ignore certain priors in its analysis. To evaluate this, we ignore priors $P_{\mathrm{hmotcr}}$, $P_{\mathrm{rdbe}}$, $P_{\mathrm{phos}},$ and $P_{\text{frag-chem}},$ see “Prior probability of the tree” section. We find that without any of these priors, the molecular formula identification rate of SIRIUS 3 drops from 76.0 to 68.7 %.

### Aligning fragmentation trees

In case a query molecule is contained in the database (spectral library) to search in, we can use any method of spectral comparison, such as peak counting or dot product, to identify the correct answer. We now want to capture the more challenging case of a search where the query molecule is *not* contained in the database. Varmuza et al. [43, 44] suggested to iterate over all molecules in the database as queries, sort all remaining database entries with respect to some similarity score, and then to evaluate the *average chemical similarity* for each query in the top *k* for $k=1,2,\ldots$. This evaluation was also used to evaluate FT alignments versus spectral comparison in [42].

But the evaluation setup suggested in [43, 44] forces a restriction upon the method that ranks the compounds in the spectral library for a given query (such methods will be called *search engine* in the following): the search engine cannot decide to return more or fewer answers for certain queries; instead, it is forced to always return the same number of answers. We argue that this restriction is somewhat artificial: even for queries where the search engine cannot find anything remotely similar to the query, the top *k* answers are nevertheless taken into consideration for evaluation.

Here, we suggest a novel evaluation setup that follows the same line of thought, but allows the search engine to return result lists of individual size. At the same time, this modification allows us to consider fractionate maximum ranks. Assume that our database *X* has size $n := |X|$. For each query $q \in X$ and each database entry $x \in X$ with $x \ne q,$ the search engine has computed some score. This results in $N := n^2 -n$ pairs $(q_1,x_1), \ldots (q_N,x_N)$ and a score for each pair. We allow the search engine to return the pairs in arbitrary order, so we sort pairs with respect to their score in descending order. We want to estimate the average chemical similarity for some fractionate rank $\kappa \ge 0$: the search engine will return the best $\kappa (n-1)$ results, rounded down. This is equivalent to saying that *on average*, the search engine will return $\kappa$ results for each query. Since we are working with reference data, we know all molecular structures and, hence, we can compute some “ground truth” chemical similarity for any pair *q*, *x*. For given $\kappa$, we can now estimate the average chemical similarity of all pairs $q_i, x_i$ for $1 \le i \le \kappa (n-1)$. An optimal search engine sorts pairs $(q_i,x_i)$ with respect to chemical similarity; this marks the best possible result that any method can achieve.

We now evaluate whether FTs computed by SIRIUS 3, together with the FT alignments from [42], result in an improved search performance compared to FTs from SIRIUS^2^-ILP combined with FT alignments. The idea of this evaluation is as follows: if the quality of SIRIUS 3 FTs is better than that of previous versions, then we may expect to also observe an improved search performance. We also evaluate against the method of estimating structural similarity using spectral comparison.

We determine chemical similarity of compounds using PubChem fingerprints and Tanimoto coefficients, as implemented in the Chemistry Development Kit (CDK) 1.5.8 [51, 52] (http://sourceforge.net/projects/cdk/). We estimate search performance both for intra-dataset and cross-database search (inter-dataset). FT similarity is computed using FT alignments as described in [42]. No optimization is performed for the FT alignment method which, in turn, was developed for FTs computed by SIRIUS^2^-DP. We also evaluate against the method of directly comparing tandem mass spectra via shared peak counting.

**Fig. 9:**
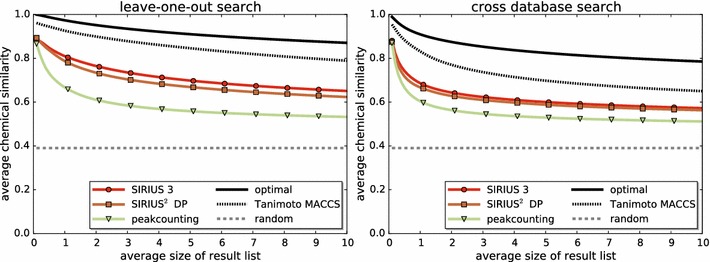
Similarity search performance plots for chemical similarity. Methods “SIRIUS 3” and “SIRIUS^2^-DP” compare trees via tree alignments [42]. Method “peak counting” uses direct spectral comparison. Method “MACCS” uses fingerprints computed from the structure of the compound. *Left* similarity search results using leave-one-out evaluation on both datasets. *Right* similarity search across databases: compounds from GNPS are searched in Agilent, and vice-versa

See Fig. 9 for the similarity search performance of these search engines. We see that FTs computed by SIRIUS 3 result in consistently improved search results for intra- and cross-database search. For the intra-database search, if the method returns 2 (5, 10, respectively) hits per query on average, then the average chemical similarity of these hits is 0.765 (0.698, 0.651) for SIRIUS 3 and, hence, about 0.03 larger than hits reported by “SIRIUS^2^-DP” trees (0.734, 0.669, 0.623). In comparison to direct spectral comparison, FT alignments improve the respective chemical similarities by up to 0.15 (0.611, 0.559, 0.533). For the inter-database search, improvements are less pronounced; we observe that all methods’ results are closer together.

“Chemical similarity” is not a well-defined measure, and there exist several approaches to estimate it. In particular, there exist other types of molecular fingerprints, such as MACCS fingerprints. We now use one type of molecular fingerprints (MACCS) to sort database compounds with respect to some query, and another type of fingerprints (PubChem) to evaluate results. This can be thought of as an upper limit of what can be reasonably reached by any method for this task. For the inter-database search, sorting compounds by their MACCS Tanimoto coefficient results in about 0.15 higher chemical similarity than the FT alignment method using SIRIUS 3. Hence, the quality difference between spectral comparison and FT alignments with SIRIUS 3, is about as large as the difference between FT alignments and the MACCS fingerprint method which has perfect knowledge about compound structure. This roughly holds true for the inter-database search, too.

We stress that we optimized neither SIRIUS 3 nor the tree alignment method from [42] for this evaluation. The improved search performance is merely a side-effect of the new scoring, and indicates the better structural quality of the new trees.

## Experimental

We use two datasets to train and evaluate SIRIUS 3. We concentrate on mass spectra in positive mode, as these constitute the majority of available spectra, and fragmentation spectra contain more peaks on average. For both datasets, we filter out compounds: for example, we discard compounds where no sufficient information is available in the database, compounds where the parent peak has large mass deviation, and large compounds. We stress that these compounds can also be analyzed with SIRIUS 3. We exclude them as only few compounds fall into these categories; the remaining compounds form more homogeneous groups that can be analyzed with identical parameters.

The GNPS dataset was downloaded from the GNPS database website at http://gnps.ucsd.edu on January 12, 2015. For 5464 compounds we download positive ion mode spectra from publicly available GNPS libraries, excluding third party libraries. We delete 327 compounds with missing or inconsistent annotations of the ion mode, as well as 254 compounds for which we do not find a PubChem identifier, and 201 compounds that are not fully connected by covalent bonds. The dataset does not comprise MS1 data, so we remove 1290 compounds where fragmentation spectra have no parent peak, as we cannot asses the mass accuracy of these spectra. Note that in application, SIRIUS 3 can process such spectra without restrictions, as the mass of the parent peak is known from the MS1 measurement. From the remaining 3392 compounds, we use 2005 where fragmentation spectra contain at least five peaks with more than 2 % relative intensity, and the mass deviation of the parent peak is below the *maximum* of 10 ppm and 2 mDa, and the ion mass is below 1000 Da. Besides CHNO, compounds contain elements sulfur (313 compounds), chlorine (250), phosphorus (13), fluorine (168), iodine (9), and bromine (14). For each compound, GNPS provides a single collision-induced dissociation (CID) fragmentation spectrum at varying collision energies, mostly recorded on an Agilent QTOF with electrospray ionization. A few compounds were measured on a different experimental platform but are not excluded. We do not correct for any mass bias during preprocessing.

The *Agilent* dataset is commercially available under the name “MassHunter Forensics/Toxicology PCDL” (version B.04.01) from Agilent Technologies Inc., (Santa Clara, CA, USA), and contains compounds of forensic and toxicological interest. In the commercial variant of the database, masses of peaks are corrected using masses of hypothetical fragments of the compound. We stress that our version of the database contains peak lists from fragmentation spectra *without any corrections* to masses or other parameters. This dataset contains 2318 compounds for which mass spectra were recorded in positive mode. We discard 53 compounds that contain deuterium, and 43 compounds that have parent mass accuracy worse than 10 ppm and five compounds with ion mass above 1000 Da. We also discard 67 compounds where the fragmentation spectrum containing <5 peaks with relative intensity 2 % or above. For 104 compounds no parent peak is recorded, so we have to exclude them from our evaluation. We end up with 2046 compounds. Besides CHNO, compounds contain elements sulfur (442 compounds), chlorine (381), phosphorus (78), fluorine (176), bromine (42), and iodine (27).

CID spectra were measured on an Agilent 6500 series instrument. For each compound, three different collision energies (10, 20, and 40 eV) were recorded. Unfortunately, only relative intensities were recorded, so we have to perform corrections to merge spectra recorded at different collision energies: we normalize each spectrum so that peak intensities sum up to one. Next, we “merge” the three spectra, and normalize the resulting peak list so that the highest peak has intensity 100 %. We then merge peaks from fragmentation spectra with different collision energies with mass difference up to 10 mDa [41]: in this case, we use the mass of the highest peak, and sum up intensities. Note that the highest peak in the resulting spectrum may have intensity above 100 %. Finally, we discard all peaks with relative intensity below 0.5 %.

It is understood that the more elements an unknown compound *may* contain, the harder it is to identify its molecular formula: the decompositions of the monoisotopic mass of the compound constitute its candidate molecular formulas, and the number of decompositions increases significantly if we consider a larger alphabet of elements. For the purpose of evaluating SIRIUS 3, we split each datasets into two disjoint batches: the batch “CHNOPS” contains all compounds that use solely elements CHNOPS. The second batch, named “contains FClBrI”, contains all compounds with at least one atom from FClBrI.

The first independent dataset was provided by Stravs et al. [49]. MS/MS data from this dataset is used without further processing or filtering. The second independent dataset consists of MS/MS spectra from MassBank [4] (http://massbank.jp). We use spectra of 1333 compounds. 958 compounds are measured on a QTOF instrument and provided by the Washington State University (266), the University of Connecticut (102), the Oswaldo Cruz Foundation (95), the Leibniz Institute for Plant Biochemistry (112), and the RIKEN Plant Science Center (383). 375 compounds are measured on a ITFT instrument and provided by the Max Planck Institute for Chemical Ecology (74), the Helmholtz Centre for Environmental Research (239), and the NAIST Graduate School of Information Science (62). We remove compounds for which we find no parent peak within 10 ppm or 2 mDa of the theoretical ion mass (252 compounds), and compounds for which the merged spectra contain <5 peaks with relative intensity over 2 % (207 compounds).

## Conclusion

We have presented a Maximum A Posteriori estimator for the problem of computing fragmentation trees, that performs significantly better than previous approaches for the problem, roughly doubling the number of correctly identified molecular formulas. Beyond estimating the hyperparameters of the method, our method SIRIUS 3 does not rely on any (spectral or structure) database. With recent methodical advances in the field [9–15], MS/MS data is increasingly searched in molecular structure databases. We argue that not depending on any databases for determining the molecular formula of an unknown compound, is an important advantage of SIRIUS 3 and any other method that does so: Restricting the search space to known molecular formulas makes the problem *much* easier and, hence, leads to favorable evaluation results for all available test data. But this approach must fail to detect compounds where not even the molecular formula is contained in any structure database.

Beyond molecular formula identification, FTs can assist in the structural elucidation of a compound, either manually or by automated means. We argue that this, in fact, is the main use of FTs; we have not discussed it in more detail here, as evaluating and comparing the performance of methods is highly elaborate and, for large-scale datasets, only possible using derived measures. As an example of an automated downstream analysis of FTs, Shen et al. [10] introduced a Machine Learning approach for determining molecular fingerprints from FTs; molecular fingerprints are then used to search molecular structure databases. Adding FTs to the prediction pipeline resulted in a significant improvement of prediction and search performance [10]. This ultimately lead to the development of the search engine CSI:FingerID (http://www.csi-fingerid.org/), which is currently the most powerful tool for searching tandem MS data in molecular structure databases [9].

Identification performance can be significantly improved by adding isotope pattern information [27, 30, 41] but this data is not available for the two datasets used here. Our evaluation assumes that isotope pattern analysis cannot provide any information on how to rank molecular formulas. We argue that this assumption is very conservative, considering that previous studies reported good identification rates using solely isotope pattern information [26, 27, 30]. Here, we deliberately use a conservative evaluation to demonstrate the power of our FT-based method in a worst-case scenario.

Different from supervised Machine Learning, hyperparameters of SIRIUS 3 are not trained in a way that maximizes, say, correct identifications of molecular formulas. Instead, hyperparameters have a statistical interpretation such as “mean loss mass”. Besides the lists of common losses and fragments, only few hyperparameters of the method are estimated from the training data. Model assumptions such as using a log-normal distribution for modeling loss masses, can be evaluated using the data. To this end, there is only a faint possibility of overfitting the method to the training data. To further rule out this possibility, we have evaluated SIRIUS 3 on an independent dataset, reaching comparable identification rates for molecular formula identification.

In our evaluations, we assume that we know in advance about the (potential) presence of “unusual elements” FClBrI. SIRIUS 3 comes with a simple classifier to predict the presence of chlorine and bromine from the data. In the near future, we will integrate a more sophisticated Machine Learning approach for this task.

We will repeat estimating the method’s hyperparameters when more training data becomes publicly available; this can further improve the method’s power in the future. In particular, when Orbitrap datasets of roughly the same size as the datasets used herein become available, we will release a version of SIRIUS that will incorporate this information.

## Methods

### Fragmentation trees

First, we will formally introduce fragmentation trees, allowing us to interpret fragmentation tree computation as a Maximum A Posteriori estimation in the next section. Our *data*${\mathcal{D}}= ({\mathcal{M}},I)$ is a measured fragmentation spectrum with peak masses ${\mathcal{M}}= \{m_1,\ldots ,m_L\}$ and peak intensities $I: {\mathcal{M}}\rightarrow {\mathbb{R}}_{> 0}$. Masses are not measured with arbitrary precision: to decide whether some theoretical molecular formula may coincide with some measured peak, we use a relative mass accuracy parameter $MA$ provided by the user. Some peak with mass *m* and a molecular formula with mass $m'$ match if $\left|m^{\prime }-m\right| \le MA \cdot m$. Usually, the mass accuracy parameter $MA$ is provided as “parts per million” (ppm); for mass accuracy 5 ppm we have $MA = 5 \times 10^{-6}$. For small masses below some threshold parameter $m < m_{ MA }$, we instead check $\left|m^{\prime }-m\right| \le MA \cdot m_{ MA }$. Fragmentation spectra are relatively sparse: for any interval of 1 Da in the spectrum, there are at most a few peaks present. On the other hand, we demand that the mass accuracy of the measurement is high, say, 20 ppm or better. To this end, almost all theoretical molecular formula can explain *at most one* peak in the measured spectrum. See below for the very rare exceptions to this rule.

A *fragmentation tree* (FT) ${\mathcal{T}}= (V,E)$ consists of a set of nodes *V* which are molecular formulas over some alphabet of elements, and directed edges (arcs) connecting these nodes. All edges are directed away from the root of the tree, and every node can be reached from the root via a unique series of edges. In small compound fragmentation, many fragments result from fragmentation cascades, that is, series of subsequent fragmentation events; these cascades are modeled by the tree structure of the FT. Nodes of the FT are molecular formulas of the parent ion and its fragments; edges correspond to losses. For any FT, each molecular formula can appear at most once as a node of the tree. For an edge $(u,v) \in E$, $u-v$ is the molecular formula of the corresponding loss; we demand that $u \ge v$ holds (for each component) and, hence, $u-v \ge 0$. Let $\mu (f)$ denote the theoretical mass of the molecular formula *f* (either fragment or loss). This will usually be the mass of the lightest naturally occurring isotope of an element, such as $\mu (H) = 1.007825$. In our calculations, we use masses from [53].

We report protonated ions as $\hbox {C}_{6}\hbox {H}_{7}\hbox {O}^{+}$ or $\hbox {C}_{6}\hbox {H}_{6}\hbox {ONa}^{+}$. We calculate masses of single-charged ions by removing a single electron mass [53, 54]. We will concentrate on protonated ions (positive mode MS); generalization to negative mode MS, as well as other forms of ionization are straightforward.

For a given FT, we can simulate a fragmentation spectrum (without intensities), simply using the masses of all nodes’ molecular formulas. For the inverse direction, a FT is supported by a fragmentation spectrum of a compound if, for every node of the tree, we find a peak in the spectrum such that the mass difference between the molecular formula of the node and the peak mass is below some user-defined threshold. Recall from the above that there can be at most one such peak. Not all peaks of the fragmentation spectrum have to be explained by the tree, as we also have to model noise peaks in the spectrum. But we demand that for every node of the FT, there is a peak in the spectrum.

By modeling the compound fragmentation as a tree, we make the implicit assumption that each fragment in the fragmentation spectrum is generated by a single fragmentation pathway. In practice, different fragmentation pathways may lead to fragments with identical molecular structure. The most prominent example is that two fragmentation events happen independently and in arbitrary order: we call this a “parallelogram” spanned by the losses *a*, *b*, and $a+b$. For the FT, we focus on the most important fragmentation process that does possibly not contain all fragmentation events, but all major fragmentation events that mainly occurred. This is a slight oversimplification of the problem, but applying the parsimony principle is necessary to formulate the task as an optimization problem. Regarding parallelograms, we note that these are implicitly encoded in the FT, as we can re-insert edges (losses) that correspond to such fragmentation events.

There is one additional requirement we need: we demand that every node of the FT explains a *unique* peak in the spectrum. In other words, no two nodes of the tree may correspond to the same peak. Allowing more than one node to explain a peak, would violate the vast majority of observations: in theory, it is possible that two fragments of a compound have different structure but very similar mass, so that both fragments explain the same peak. In practice, this situation is extremely rare, and excluding this “pathological” cases is again necessary to formulate our task as an optimization problem: the improvement by making this assumption outweighs the cases where it leads to a possible incorrect interpretation.

We now formalize our above considerations. We say that a FT ${\mathcal{T}}= (V,E)$ is *supported by* the observed data ${\mathcal{D}}= ({\mathcal{M}},I)$ if each node $v \in V$ is assigned a unique peak $m \in {\mathcal{M}}$ in the fragmentation spectrum that is within the chosen mass accuracy. Furthermore, no two nodes are assigned the same peak. We denote the *natural injective mapping* from the FT nodes to the peaks by $m: V \rightarrow {\mathcal{M}}$. All peaks in the spectrum not assigned to a node of the FT, are regarded as noise peaks. Our task is to find a FT that “best explains” the observed data, where goodness-of-fit is measured by some scoring function (such as the posterior probability estimate considered below) that matches FT and mass spectrum.

This formulation of the problem is not easily accessible by algorithmic means; to this end, we use an alternative formulation which, for additive scorings, is equivalent to the above [34]: for each peak in the fragmentation spectrum, we find all molecular formulas with mass difference sufficiently small. These molecular formulas are the nodes of a directed acyclic graph (DAG) called *fragmentation graph*. Nodes are colored so that all molecular formulas corresponding to the same peak have the same color. Recall that we must use at most one vertex for each color (peak) in our FT. Edges are inserted whenever one molecular formula is a sub-formula of another. Edges are appropriately weighted using some score function. It is straightforward to check that there is a 1–1 correspondence between *colorful* subtrees, that use every color in the graph at most once, and FTs supported by the data. We search for a colorful subtree of this graph that has maximum weight.

To identify the molecular formula of the unknown compound, we can add a super-root that is connected to all molecular formula explanations of the parent ion peak. As all of the corresponding nodes share the same color, only one interpretation of the parent ion peak will be present in the optimal solution. In practice, it turns out to be faster to instead consider one molecular formula for the parent ion peak at a time, compute for each such candidate an optimal FT, and rank the resulting trees according to their posterior probability.

We have deliberately ignored that the mass difference between two measured peaks in ${\mathcal{D}}$ may be smaller than twice the chosen mass accuracy; in this case, two peaks would be assigned the same molecular formula in the fragmentation graph and, possibly, also the maximum colorful subtree, violating our condition that all nodes have to be different molecular formulas. In practice, this situation will show up extremely rarely for mass accuracy of 10 ppm or better. If this “pathological” situation turns up, we split the mass range between the two measured peaks in half, so that any molecular formula is forced towards the closer measured peak.

### Maximum A Posteriori estimation

Scorings in [34, 41, 42] were motivated by stochastic considerations, but only in an informal way. Here, we will strictly model the problem as a Maximum A Posteriori estimation, which allows us to make sensible choices for the (hyper)parameters of the method. Bayesian Statistics tell us that

1 $${\mathbb{P}}({\mathcal{T}}_j | {\mathcal{D}}) = \frac{{\mathbb{P}}({\mathcal{D}}| {\mathcal{T}}_j) \cdot {\mathbb{P}}({\mathcal{T}}_j)}{{\mathbb{P}}({\mathcal{D}})} = \frac{{\mathbb{P}}({\mathcal{D}}| {\mathcal{T}}_j) \cdot {\mathbb{P}}({\mathcal{T}}_j)}{\sum \nolimits _i {\mathbb{P}}({\mathcal{D}}| {\mathcal{T}}_i) \, {\mathbb{P}}({\mathcal{T}}_i)},$$

where ${\mathcal{D}}$ is the data (the measured spectrum) and ${\mathcal{T}}_j$ are the models (the candidate FTs). We want to maximize the posterior probability ${\mathbb{P}}({\mathcal{T}}_j | {\mathcal{D}})$ which is equivalent to maximizing ${\mathbb{P}}({\mathcal{D}}| {\mathcal{T}}) \cdot {\mathbb{P}}({\mathcal{T}})$ over all possible models ${\mathcal{T}}$. Here, ${\mathbb{P}}({\mathcal{D}}| {\mathcal{T}})$ is the probability of the data given the model ${\mathcal{T}}$, and ${\mathbb{P}}({\mathcal{T}})$ is the *prior probability* of model ${\mathcal{T}}$, based on prior information that we have about FTs without considering the actual data ${\mathcal{D}}$. We have considerable background information about the prior probability of any given FT: for example, smaller losses are usually more frequent than larger losses for low and medium energy fragmentation, and certain losses such as $\hbox {H}_{2}\hbox {O}$ or CO turn up very frequently.

We have stressed repeatedly that we are interested in those FTs only that are supported by the data. To this end, we demand ${\mathbb{P}}({\mathcal{D}}|{\mathcal{T}}) = 0$ and, hence, ${\mathbb{P}}({\mathcal{T}}|{\mathcal{D}}) = 0$ for any tree ${\mathcal{T}}$ that is *not supported by the data* ${\mathcal{D}}$. In the following, we assume that each considered FT is supported by the data.

We now introduce computations for prior probability and likelihood of the tree.

### Prior probability of the tree

We first concentrate on the prior ${\mathbb{P}}({\mathcal{T}})$. We will not demand that priors sum to one but only that the sum $\sum \nolimits _i {\mathbb{P}}({\mathcal{T}}_i) \, {\mathbb{P}}({\mathcal{D}}| {\mathcal{T}}_i)$ converges, what is sufficient for optimizing ${\mathbb{P}}({\mathcal{T}}) \cdot {\mathbb{P}}({\mathcal{D}}| {\mathcal{T}})$. But this is obviously true: the number of models ${\mathcal{T}}_i$ we are considering is finite, as we are only consider trees supported by the data. We assume that, for all trees of constant size, prior probabilities of the nodes and edges of ${\mathcal{T}}$ are independent so that

$${\mathbb{P}}({\mathcal{T}}) = {\mathbb{P}}(\text{size}\, \left|E\right|\, \text{of the tree}) \cdot \prod \limits _{v \in V} {\mathbb{P}}(v) \cdot \prod \limits _{e \in E} {\mathbb{P}}(e) .$$

where ${\mathbb{P}}(v)$ is the prior probability to see a particular *fragment* in a FT, and ${\mathbb{P}}(e)$ is the prior probability to see a particular *loss* in a FT. The independence assumption is obviously violated in reality, but allows us to come up with simple yet meaningful priors. We can simplify this equation, noting that every node of the tree except the root has exactly one incoming edge. For molecular formulas *u*, *v* let $P_{\mathrm{edge}}(u,v)$ be the prior that fragment *vand* loss $u-v$ are simultaneously seen in the tree, and let $P_{\mathrm{root}}(u)$ be the prior that the tree is rooted with molecular formula *u*. Then,

2 $$\begin{aligned}&{\mathbb{P}}({\mathcal{T}}) \varpropto {\mathbb{P}}(\text{size}\, \left|E\right|\, \text{of the tree})\\ &\quad \cdot P_{\mathrm{root}}(r) \cdot \prod _{(u,v) \in E} P_{\mathrm{edge}}(u,v)\end{aligned}$$

where *r* is the root of ${\mathcal{T}}$.

#### Prior of the root

For the prior $P_{\mathrm{root}}(r)$ of the root *r* we use the molecular formula *r*, and the fact that certain molecular formulas are observed more often in molecular databases [20]. We use the following uninformative prior to filter out structurally impossible molecular formulas: for each compound, the sum of valences has to be greater than or equal to twice the number of atoms minus one; this is one of the “Senior rules” [55]. This corresponds to a non-negative ring double bond equivalent (RDBE) value, which is defined as

3 $$\begin{aligned} \text{RDBE} &= 1 + \tfrac{1}{2} \left( 2 \#\text{C} - \#\text{H}\right.\\ &\left.\quad + \#\text{N} + \#\text{P} - \#\text{Cl} - \#\text{Br} -\#\text{I} - \#\text{F}\right)\end{aligned}$$

where $\#\text{E}$ denotes the number of atoms for element E. There exist some exceptions to this rule [20]; if the molecular formulas of such compounds is to be determined, this uninformative prior has to be modified.

In addition, we use three informative priors suggested previously [20, 34, 41], all of which apply for the root only. For the rest of this section, we will consider molecular formulas from the Kyoto Encyclopedia of Genes and Genomes (KEGG) [56]. This database contains 17,529 molecular structures of metabolites, and we will consider it as a “uniform subsample” of all possible such biomolecules.

First, assume that the compound is not a radical, then the sum of valences is even [55]. If the compound ion is protonated or has its charge due to some adduct, then the sum of valences of the ion is odd. Rejecting compounds with uneven sum of valences is also referred to as a “Senior rule” [55]. But certain compounds are intrinsically charged; for these compounds, the sum of valences is even. Also, free radicals such as nitrosyls have, in their protonated form, even sum of valences. But both intrinsically charged molecules and free radicals are comparatively rare; to this end, we use $P_{\text{rdbe-odd}} = 0.1$ for molecular formulas with even sum of valences, and $P_{\text{rdbe-odd}} = 1$ for all others.

**Fig. 10:**
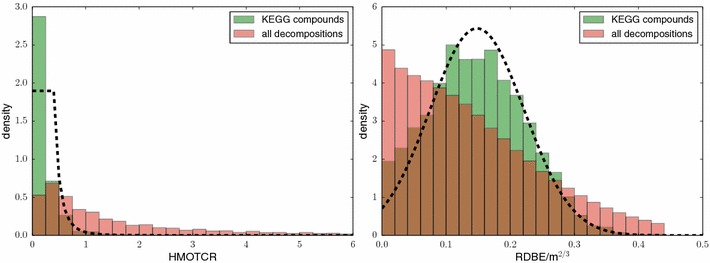
*Left* histogram of compounds from KEGG that show a particular ratio of hetero atoms except oxygen, and carbon atoms (*green*); histogram of all decompositions of compound masses from KEGG over the alphabet CHNOPS with mass accuracy 10 ppm (*red*). We observe that compounds from KEGG [56] have relatively small ratios, whereas this ratio can get arbitrarily large for the decompositions that, in most cases, do not correspond to true molecules. Normalized density of the prior (*dashed*). *Right* histogram of the corrected RDBE values from (4) (*green*); histogram of all decompositions (*red*); normalized density of the prior (*dashed*)

Second, the ratio between hetero atoms and carbon atoms is usually relatively small for biomolecules [20]. We find that this ratio becomes even more informative if we also exclude oxygen from the hetero atoms, see Fig. 10. We model the “hetero minus oxygen to carbon ratio” (HMOTCR) using a uniform prior $P_{\mathrm{hmotcr}} = 1.8969$ for ratios in [0, 0.4];  for ratios above 0.4, $P_{\mathrm{hmotcr}}$ follows a Pareto distribution with $x_{\min} = 0.4$ and $\alpha = 3.1453.$

Third, the ring double bond equivalent (RDBE) values can be used as a prior [20]: we observe that the value

4 $$\text{corrected RDBE} = \frac{\text{RDBE}}{m^{2/3}} ,$$

where *m* is the mass of the compound, is roughly normal distributed, see Fig. 10. We use the density of the normal distribution with $\mu = 0.1482$, $\sigma = 0.0734$ as the prior $P_{\mathrm{rdbe}}(r)$ for the corrected RDBE value.

We add a fourth prior for penalizing molecular formulas containing “special” elements. We define all elements but C, H, N, O as *special*, as they occur less often in metabolites and natural products. We define $P_{\mathrm{spec}} = 0.25^{n}$ with *n* being the number of special elements in the molecular formula. We stress that this is *not the number of atoms* corresponding to special elements: for example, $\hbox {C}_{17}\hbox {H}_{17}\hbox {C}_{l2}\hbox {N}$ has $n=1$. The basic idea behind this prior is to penalize the occurrence of special elements in the molecular formula if there are no further indications (in losses or fragments) for this element. We later add other edge and node priors to counter the effect of the special elements prior.

Additionally, we add a prior for penalizing phosphorus-containing molecular formulas without oxygen or sulfur: we find that for more than 99 % of the phosphorus-containing compounds in the KEGG metabolite database, the sum of oxygen and sulfur atoms is at least twice the number of phosphorus atoms. We set $P_{\mathrm{phos}} = 0.05$ for all compounds that violate this constraint, and $P_{\mathrm{phos}}= 1$ otherwise.

The root prior

$$\begin{aligned} P_{\mathrm{root}}(r)&= {} P_{\text{rdbe-odd}}(r) \cdot P_{\mathrm{hmotcr}}(r)\nonumber \\&\quad \cdot P_{\mathrm{rdbe}}(r) \cdot P_{\mathrm{spec}}(r) \cdot P_{\mathrm{phos}}(r) \end{aligned}$$

is the product of these five priors. We stress that informative priors never discard any molecular formulas but rather, decrease the likelihood of these formulas.

We do not use additional priors as suggested in [20, 34, 41]. We found that these additional priors do not worsen results, but also do not lead to any improvement worth mentioning.

#### Priors of edges

The prior probability $P_{\mathrm{edge}}(u,v)$ of an edge $e = (u,v)$ is estimated from different factors, namely prior knowledge about implausible (and radical) losses, the mass of the loss, common losses, as well as common fragments:

5 $$\begin{aligned} P_{\mathrm{edge}}(u,v)&:= {} P_{\text{loss-impl}}(u,v) \cdot P_{\text{loss-mass}}(u,v) \cdot P_{\text{loss-comm}}(u,v)\nonumber \\ &\quad \cdot P_{\text{loss-spec}}(u,v)\cdot P_{\text{frag-chem}}(u,v)\\ &\quad \cdot P_{\text{frag-mass}}(v) \cdot P_{\text{frag-comm}}(v) \end{aligned}$$

We first penalize *implausible losses* of an edge (*u*, *v*) using a prior $P_{\text{loss-impl}}(u,v)$ on the loss $u-v$. This is a small list of losses that repeatedly turned up during our combinatorial optimization in [41], but that were rejected in the subsequent expert evaluation given there. In particular, we penalize losses that contain only nitrogen or only carbon; radical losses with certain exceptions; and few losses from a list of losses generated by expert knowledge. See Table 2 for the list of implausible losses and priors. Since these are losses that we *do not want to see*, there appears to be no reasonable way to learn such implausible losses from the data. Instead, we have to rely on expert knowledge and evaluation of FTs computed by the method, to collect this list. Also, priors for such implausible losses are chosen ad hoc as there appears to be no sensible way of learning such penalties from the data.

**Table 2:** Priors for *implausible losses*

Probability	Loss type and molecular formulas
$10^{-3}$	Implausible losses: $\hbox {C}_{2}\hbox {O}$, $\hbox {C}_{4}\hbox {O}$, $\hbox {C}_{3}\hbox {H}_{2}$, $\hbox {C}_{5}\hbox {H}_{2}$, $\hbox {C}_{7}\hbox {H}_{2}$
$\frac{1}{3^{ rdbe }}$	Neutral losses with negative ring double bond equivalent RDBE
0.1	Nitrogen-only losses, carbon-only losses: for example, $\hbox {N}_{5}$ or $\hbox {C}_{3}$
1	All other neutral losses
0.9	Common radical losses: $\hbox {H}^{\cdot }$, $\hbox {O}^{\cdot }$, ${}^{\cdot }\hbox {OH}$, ${}^{\cdot }\hbox {CH}_{3}$, $\hbox {CH}_{3}\hbox {O}^{\cdot }$, ${}^{\cdot }\hbox {C}_{3}\hbox {H}_{7}$, ${}^{\cdot }\hbox {C}_{4}\hbox {H}_{9}$, $\hbox {C}_{6}\hbox {H}_{5}\hbox {O}^{\cdot }$
$10^{-3}$	All other radical losses

For an edge (*u*, *v*) with loss $u-v$ let $P_{\text{loss-impl}}(u,v)$ be the prior for $u-v$ chosen according to this table. Expert knowledge and evaluation of FTs from SIRIUS^2^ resulted in the implausible losses listed here [41]. These losses should only very rarely (if ever) occur in a FT, so we manually select reduced priors

Regarding the mass of a loss, we assume that large losses are less likely than small losses. Unfortunately, there is only a very small number of annotated FTs available in the literature, and these are usually measured on different instruments (and instrument types) using different experimental setup and, hence, mostly incomparable. To this end, we estimate the loss mass distribution using FTs determined by SIRIUS 3. We will try to bring in agreement the observed distributions with the distribution used for scoring.

Different from [34, 41, 42] we do not penalize the relative size of the mass but rather the mass itself, as this allows for a more stringent incorporation of common losses. Combinatorics dictates that there exists only a small number of losses below, say, 30 Da. Besides certain common losses, this implies that the number of small losses is also small, but increases rapidly until some maximum is reached. Beyond this mass, we find that the probability to observe a loss drops rapidly in the beginning, but stays significantly above zero even for large masses. To model these observations, we use a log-normal distribution as a classical example of a long-tailed distribution. Let $\mu _{\text{ls}}, \sigma _{\text{ls}}$ be the parameters of the log-normal distribution, then the probability density function is

$$\tfrac{1}{x \sqrt{2\pi \sigma _{\text{ls}}^2}} \, \exp \left( -\tfrac{\left( \ln x-\mu _{\text{ls}}\right) ^2}{2\sigma _{\text{ls}}^2} \right)$$

for mass *x*. See “Statistics and fitting the model” section for the fitting of hyperparameters $\mu _{\text{ls}}, \sigma _{\text{ls}}$; there, we report an excellent fit of loss masses using the log-normal distribution. We use

6 $$P_{\text{loss-mass}}(\Delta ) := \tfrac{1}{\Delta \sqrt{2\pi \sigma _{\text{ls}}^2}} \, \exp \left( -\tfrac{\left( \ln \Delta -\mu _{\text{ls}}\right) ^2}{2\sigma _{\text{ls}}^2} \right)$$

for mass delta $\Delta > 0$ as the loss mass prior, and set $P_{\text{ loss-mass}}(u,v) := P_{\text{ loss-mass}}(\mu (u-v))$.

Some losses turn up more often than we would expect from the loss mass distribution. Instead of relying on an expert-curated list we learn common losses and their prior probabilities from our training data, see “Statistics and fitting the model” section; and see Table 3 for the actual priors $P_{\text{loss-comm}}(u,v).$

**Table 3:** Priors $P_{\text{loss-comm}}(l)$ for *common losses* *l*

Mol. formula	Mass	Loss name	Known	Intensity GNPS		Intensity Agilent		$P_{\text{loss-comm}}$
				Total	Expected	Total	Expected	
H^a^	1.0078	Hydrogen radical		110	0.00	77	0.00	^a^
$\hbox {H}_{2}^{\mathrm{a}}$	2.0157	Hydrogen	A, B	1799	0.00	890	0.00	^a^
$\hbox {CH}_{2}$	14.0157	Methylene		33	17.47	71	37.35	1.92
$\hbox {CH}_{3}$	15.0235	Methyl	A	3231	46.48	1481	21.31	69.53
$\hbox {CH}_{4}$	16.0313	Methane	A, B, C	2011	75.23	929	34.76	26.73
$\hbox {H}_{3}\hbox {N}$	17.0265	Ammonia	A, B, C	1409	62.73	1481	65.92	22.47
$\hbox {H}_{2}\hbox {O}$	18.0106	Water	A, B, C	5548	85.53	4014	61.88	64.87
HF	20.0062	Hydrogen fluoride		266	13.43	365	18.36	19.88
$\hbox {C}_{2}\hbox {H}_{2}$	26.0157	Ethine	B, C	2434	133.98	2324	127.90	18.17
CHN	27.0109	Hydrogen cyanide		1117	139.90	1078	134.94	7.99
CO	27.9949	Carbon monoxide	B, C	4232	177.14	2614	109.45	23.89
$\hbox {C}_{2}\hbox {H}_{4}$	28.0313	Ethene	A, B, C	483	87.19	1108	199.82	5.55
$\hbox {CH}_{3}\hbox {N}$	29.0265	Methyleneimine	B	347	158.43	305	139.34	2.19
S	31.9721	Sulfur	B, C	79	38.60	179	87.07	2.06
$\hbox {CH}_{4}\hbox {O}$	32.0262	Methyl esters		202	127.42	341	214.18	1.59
Cl	34.9689	Chlorine		296	45.18	394	60.27	6.55
HCl	35.9767	Hydrogen chloride		462	45.88	613	60.95	10.07
$\hbox {C}_{2}\hbox {H}_{2}\hbox {O}$	42.0106	Ketene	B, C	811	246.67	584	177.75	3.29
$\hbox {C}_{3}\hbox {H}_{6}$	42.0470	Propene		207	101.85	656	322.40	2.03
$\hbox {C}_{2}\hbox {H}_{5}\hbox {N}$	43.0422	Aminoethylene		332	177.18	454	242.22	1.88
$\hbox {CO}_{2}$	43.9898	Carbon dioxide	B, C	281	199.41	215	153.06	1.41
Br	78.9183	Bromine		20	0.91	95	4.23	22.51
HBr	79.9262	Hydrogen bromide		9	0.63	65	4.38	14.98
$\hbox {HO}_{3}\hbox {P}$	79.9663	Metaphosphoric acid	B, C	3	0.78	25	6.55	3.93
$\hbox {HO}_{2}\hbox {PS}$	95.9435	Phosphenothioic acid		0	0.11	26	4.60	5.65
I	126.9045	Iodine		29	0.25	60	0.52	116.53
HI	127.9123	Hydrogen iodide		11	0.15	45	0.61	74.61
CIO	154.8994	Iodomethanone		0	0.04	3	0.32	10.28
$\hbox {C}_{10}\hbox {H}_{9}\hbox {NO}_{3}\hbox {S}^{\mathrm{b}}$	223.0303			20	1.12	5	0.30	18.54
$\hbox {C}_{12}\hbox {H}_{8}\hbox {ClNS}^{\mathrm{b}}$	233.0066	2-Chlorophenothiazine		1	0.06	25	0.83	30.72
$\hbox {I}_{2}$	253.8089	Iodine		0	0.00	10	0.03	357.31
$\hbox {C}_{11}\hbox {H}_{10}\hbox {Cl}_{2}\hbox {N}_{2}\hbox {O}^{\mathrm{b}}$	256.0170			3	0.12	9	0.40	24.93

Entry “mass” is the exact theoretical mass of the loss. Entry “known” indicates whether the loss was included in the expert-curated common loss lists in A [34], B [41], or C [42]. Entry “total” indicate the (rounded) frequency of the loss in the trees computed from the dataset, weighted by the maximum peak intensity of the two peaks that are responsible for this loss. Entries “expected” is the weighted frequency we would expect from the loss mass prior, and $P_{\text{loss-comm}}$ is the common loss prior after correcting for the loss mass prior

^a^Losses H and $\hbox {H}_{2}$ can be interpreted as artifacts of the loss mass prior

^b^ $\hbox {C}_{10}\hbox {H}_{9}\hbox {NO}_{3}\hbox {S}$, $\hbox {C}_{12}\hbox {H}_{8}\hbox {ClNS}$ and $\hbox {C}_{11}\hbox {H}_{10}\hbox {Cl}_{2}\hbox {N}_{2}\hbox {O}$ are artifacts, stemming from either their high mass or the small number of chlorine-containing compounds in the datasets

The $P_{\text{loss-spec}}$ prior counters the effect of the $P_{\mathrm{spec}}$ root prior. We observe that common losses and low mass peaks are reliable indicators for the presence of special elements in the compound. We set $P_{\text{loss-spec}} = 1.5$ for all fragments for which either the incoming edge (loss) is a common loss containing a special element, or for which the fragment itself contains a special element and has mass below 75 Da.

For a FT to be informative, it is useful that the FT includes fragments of small masses, even if the corresponding peaks have small intensities and, possibly as a result, larger mass deviations. In addition, one can relatively easily identify the fragment’s correct molecular formula, as well as distinguish fragment peaks from noise, due to the small “combinatorial diversity”: the chance that the mass of a noise peak coincidence with the theoretical mass of a molecular formula is very small for small masses. As a theoretical example, consider masses below 15 Da: in this mass region, reasonable molecular formulas are H, $\hbox {H}_{2},$ CH and $\hbox {CH}_{2}.$ To this end, all peaks with other masses *must* be noise. The fragment mass prior favors peaks with small masses,

$$\begin{aligned} P_{\text{frag-mass}}(u) = \left\{ \begin{array}{ll} 1 &{}\quad \text{ if }\;m(u) > 200 \\ e^{2 \frac{m(u)}{200}} &{}\quad \text{ otherwise, }\end{array}\right. \end{aligned}$$

to encourage the integration of small peaks that allow for a mass decomposition. The threshold of 200 Da has been chosen *ad hoc* and without any further optimization; we expect that choosing, say, a threshold of 100 Da will not result in significant differences.

We use both the hetero minus oxygen to carbon ratio (HMOTCR) and the RDBE value of a fragment *v* in comparison to its parent *u*. As proposed in [34] we do not penalize a child if we have already penalized the parent, as both HMOTCR and RDBE values are hereditary. To this end, we use

$$P_{\text{frag-chem}}(u,v) = \min \left\{ 1, \frac{P_{\mathrm{hmotcr}}(v)}{P_{\mathrm{hmotcr}}(u)} \cdot \frac{P_{\mathrm{rdbe}}(v)}{P_{\mathrm{rdbe}}(u)} \right\} .$$

Finally, we notice that certain fragments turn up repeatedly in FTs. The explanation for this observation is simple and is known to MS experts for decades: certain groups such as $\hbox {C}_{6}\hbox {H}_{5}^{+}$ (benzyne) or $\hbox {C}_{4}\hbox {H}_{8}\hbox {N}^{+}$ (pyrroline) can be cleaved off as ions, leading to characteristic peaks in the mass spectra. But giving priors for both common losses *and* common fragments, clearly violates the independence assumption: if we know the molecular formulas of a fragment and one of its losses, then this also tells us the molecular formula of the child fragment. To this end, we use a “cautious” prior that rewards only few and small common fragments which are observed very often, whereas the vast majority of fragments receive a flat prior. See “Statistics and fitting the model” section for how we learn the common fragments and their priors from the data; and see Table 4 for the actual priors $P_{\text{frag-comm}}(u,v).$

**Table 4:** Priors $P_{\text{frag-comm}}(f)$ for *common fragments* *f*

Molecular formula		Ion mass	Total intensity		Total count		$P_{\text{frag-comm}}$
Protonated	Neutral		GNPS	Agilent	GNPS	Agilent	
C_3_H_6_N^+^	C_3_H_5_N	56.0495	0.00	93.63	0	392	2.40
C_3_H_8_N^+^	C_3_H_7_N	58.0651	0.67	100.53	4	323	2.59
$\hbox {C}_{5}\hbox {H}_{5}^{+}$	C_5_H_4_	65.0386	0.00	83.35	0	530	2.14
$\hbox {C}_{4}\hbox {H}_{8}\hbox {N}^{+}$	$\hbox {C}_{4}\hbox {H}_{7}\hbox {N}$	70.0651	7.38	56.00	8	313	1.62
$\hbox {C}_{4}\hbox {H}_{10}\hbox {N}^{+}$	$\hbox {C}_{4}\hbox {H}_{9}\hbox {N}$	72.0808	0.00	72.92	0	179	1.87
$\hbox {C}_{6}\hbox {H}5^{+}$	$\hbox {C}_{6}\hbox {H}_4$	77.0386	1.00	139.35	3	720	3.60
$\hbox {C}_{6}\hbox {H}_{7}^{+}$	$\hbox {C}_{6}\hbox {H}_{6}$	79.0542	0.52	69.85	3	514	1.80
$\hbox {C}_{5}\hbox {H}_{12}\hbox {N}^{+}$	$\hbox {C}_{5}\hbox {H}_{11}\hbox {N}$	86.0964	0.92	71.08	5	128	1.85
$\hbox {C}_{7}\hbox {H}_{7}^{+}$	$\hbox {C}_{7}\hbox {H}_{6}$	91.0542	60.61	252.97	300	720	8.04
$\hbox {C}_{6}\hbox {H}_{6}\hbox {N}^{+}$	$\hbox {C}_{6}\hbox {H}_{5}\hbox {N}$	92.0495	3.92	76.12	31	185	2.05
$\hbox {C}_{6}\hbox {H}_{9}\hbox {O}^{+}$	$\hbox {C}_{6}\hbox {H}_{8}\hbox {O}$	97.0648	10.95	58.00	37	86	1.77
$\hbox {C}_{6}\hbox {H}_{12}\hbox {N}^{+}$	$\hbox {C}_{6}\hbox {H}_{11}\hbox {N}$	98.0964	8.73	74.93	66	139	2.14
$\hbox {C}_{8}\hbox {H}_{7}^{+}$	$\hbox {C}_{8}\hbox {H}_{6}$	103.0542	64.49	34.61	562	241	2.54
$\hbox {C}_{7}\hbox {H}_{5}\hbox {O}^{+}$	$\hbox {C}_{7}\hbox {H}_{4}\hbox {O}$	105.0335	50.43	47.64	178	100	2.51
$\hbox {C}_{8}\hbox {H}_{9}^{+}$	$\hbox {C}_{8}\hbox {H}_{8}$	105.0699	108.25	104.51	580	352	5.45
$\hbox {C}_{7}\hbox {H}_{7}\hbox {O}^{+}$	$\hbox {C}_{7}\hbox {H}_{6}\hbox {O}$	107.0491	62.41	53.68	320	187	2.98
$\hbox {C}_{8}\hbox {H}_{11}^{+}$	$\hbox {C}_{8}\hbox {H}_{10}$	107.0855	35.23	29.89	171	120	1.67
$\hbox {C}_{6}\hbox {H}_{6}\hbox {NO}^{+}$	$\hbox {C}_{6}\hbox {H}_{5}\hbox {NO}$	108.0444	23.05	48.66	64	76	1.84
$\hbox {C}_{7}\hbox {H}_{9}\hbox {O}^{+}$	$\hbox {C}_{7}\hbox {H}_{8}\hbox {O}$	109.0648	37.15	53.40	161	107	2.32
$\hbox {C}_{9}\hbox {H}_{7}^{+}$	$\hbox {C}_{9}\hbox {H}_{6}$	115.0542	73.68	43.35	618	262	3.00
$\hbox {C}_{9}\hbox {H}_{9}^{+}$	$\hbox {C}_{9}\hbox {H}_{8}$	117.0699	61.67	46.66	371	206	2.78
$\hbox {C}_{9}\hbox {H}_{11}^{+}$	$\hbox {C}_{9}\hbox {H}_{10}$	119.0855	51.94	40.94	265	190	2.38
$\hbox {C}_{8}\hbox {H}_{9}\hbox {O}^{+}$	$\hbox {C}_{8}\hbox {H}_{8}\hbox {O}$	121.0648	125.09	72.05	394	201	5.05
$\hbox {C}_{10}\hbox {H}_{8}^{+}$	$\hbox {C}_{10}\hbox {H}_{7}$	128.0621	51.79	13.98	305	98	1.69
$\hbox {C}_{10}\hbox {H}_{9}^{+}$	$\hbox {C}_{10}\hbox {H}_{8}$	129.0699	60.60	24.99	425	163	2.19
$\hbox {C}_{9}\hbox {H}_{8}\hbox {N}^{+}$	$\hbox {C}_{9}\hbox {H}_{7}\hbox {N}$	130.0651	75.54	31.39	343	159	2.74
$\hbox {C}_{10}\hbox {H}_{11}^{+}$	$\hbox {C}_{10}\hbox {H}_{10}$	131.0855	61.12	34.37	277	144	2.45
$\hbox {C}_{9}\hbox {H}_{10}\hbox {N}^{+}$	$\hbox {C}_{9}\hbox {H}_{9}\hbox {N}$	132.0808	58.68	21.70	216	99	2.06
$\hbox {C}_{8}\hbox {H}_{7}\hbox {O}_{2}^{+}$	$\hbox {C}_{8}\hbox {H}_{6}\hbox {O}_2$	135.0441	40.37	22.03	176	53	1.60
$\hbox {C}_{9}\hbox {H}_{11}\hbox {O}^{+}$	$\hbox {C}_{9}\hbox {H}_{10}\hbox {O}$	135.0804	42.95	32.87	221	121	1.94
$\hbox {C}_{11}\hbox {H}_{11}^{+}$	$\hbox {C}_{11}\hbox {H}_{10}$	143.0855	54.61	23.88	288	118	2.01
$\hbox {C}_{10}\hbox {H}_{10}\hbox {N}^{+}$	$\hbox {C}_{10}\hbox {H}_{9}\hbox {N}$	144.0808	61.59	20.67	220	99	2.11
$\hbox {C}_{11}\hbox {H}_{13}^{+}$	$\hbox {C}_{11}\hbox {H}_{12}$	145.1012	57.60	28.15	219	110	2.20
$\hbox {C}_{9}\hbox {H}_{8}\hbox {NO}^{+}$	$\hbox {C}_{9}\hbox {H}_{7}\hbox {NO}$	146.0600	62.09	7.40	242	52	1.78
$\hbox {C}_{10}\hbox {H}_{11}\hbox {O}^{+}$	$\hbox {C}_{10}\hbox {H}_{10}\hbox {O}$	147.0804	67.16	33.97	247	107	2.59
$\hbox {C}_{11}\hbox {H}_{11}\hbox {O}^{+}$	$\hbox {C}_{11}\hbox {H}_{10}\hbox {O}$	159.0804	47.36	17.07	230	84	1.65
$\hbox {C}_{10}\hbox {H}_{10}\hbox {NO}^{+}$	$\hbox {C}_{10}\hbox {H}_{9}\hbox {NO}$	160.0757	58.53	18.10	221	40	1.96
$\hbox {C}_{13}\hbox {H}_{9}^{+}$	$\hbox {C}_{13}\hbox {H}_{8}$	165.0699	54.17	36.32	255	163	2.32
$\hbox {C}_{13}\hbox {H}_{11}^{+}$	$\hbox {C}_{13}\hbox {H}_{10}$	167.0855	28.57	36.85	123	65	1.68
$\hbox {C}_{12}\hbox {H}_{11}\hbox {O}^{+}$	$\hbox {C}_{12}\hbox {H}_{10}\hbox {O}$	171.0804	44.97	28.37	164	62	1.88

Entry “ion mass” is the exact theoretical mass of the protonated fragment. Entries “GNPS/Agilent” indicate total sum of the peak intensities and total peak count of the fragment in the two datasets. Note that a particular fragment can be very common, yet have relatively small sum of peak intensities, because fragments peaks are consistently of small intensity

#### Prior of the tree size

The FT we will compute should explain a large number of peaks; to this end, we want to favor large trees over small ones. The priors we have introduced so far do exactly the opposite: many edges result in many probabilities we have to multiply, and small trees are favored over large trees. To this end, we introduce one last prior: we assume

7 $$\begin{aligned}&{\mathbb{P}}\left( \text{size }\left|E\right|\text{ of the tree}\right) \varpropto P_{\text{tree-size}}^{\left|E\right|}\nonumber \\&\quad \text{where} \quad P_{\text{tree-size}} := P_{\text{tree-norm}} \cdot P_{\text{tree-bonus}}. \end{aligned}$$

where $P_{\text{tree-norm}}$ is chosen to counter the effects of the other priors on average, whereas $P_{\text{tree-bonus}}$ can be set by the user to favor smaller or larger trees. See “Statistics and fitting the model” section for how an appropriate default value of this prior is estimated from data.

### Likelihood of the tree

Recall that each considered FT ${\mathcal{T}}= (V,E)$ is supported by the data ${\mathcal{D}}= ({\mathcal{M}},I)$. This implies the existence of a natural injective mapping $m: V \rightarrow {\mathcal{M}}$: each node $v \in V$ is assigned a unique peak *m*(*v*) in the fragmentation spectrum. All peaks in the spectrum not assigned to a node of the FT, are noise peaks and also contribute to the likelihood of the tree. Also recall that each node $v \in V$ is the molecular formula of the corresponding hypothetical fragment, whereas an edge (*u*, *v*) corresponds to a loss $v-u$.

To simplify our computations, we assume independence between the measured peaks in ${\mathcal{M}}= \{m_1,\ldots ,m_L\}$:

$${\mathbb{P}}({\mathcal{D}}| {\mathcal{T}}) = \prod _l {\mathbb{P}}\left( m_l | {\mathcal{T}}\right)$$

This simplifying assumption implies that mass deviations and intensities of the individual peaks are independent of each other. Such independence assumptions are commonly used to make a stochastical model computable. Here and in the following, $m_l$ refers both to the *l*th peak and to its mass. Furthermore, we may assume that for each peak, the probability of the tree to generate some peak depends only on the corresponding hypothetical fragment, so ${\mathbb{P}}(m(v) | {\mathcal{T}}) = {\mathbb{P}}(m(v) | v)$ for all $v \in V$. Then,

$$\begin{aligned} {\mathbb{P}}({\mathcal{D}}| {\mathcal{T}})&= {} \prod \limits _l {\mathbb{P}}(m_l | {\mathcal{T}}) = \prod \limits _{v \in V} {\mathbb{P}}(m(v) | v)\\&\quad \cdot {\mathbb{P}}(\text{unassigned peaks} | {\mathcal{T}}) \end{aligned}$$

for appropriately chosen ${\mathbb{P}}(m(v) | v)$. Here, ${\mathbb{P}}(\text{unassigned peaks} | {\mathcal{T}})$ is the probability that all unassigned peaks ${\mathcal{M}}- \{m(v) :v \in V\},$ which cannot be explained by ${\mathcal{T}}$, are noise peaks.

Unassigned peaks cannot be scored in the FT optimization, as only those nodes and edges are scored that are actually part of the tree. To get rid of the probability of unassigned peaks, note again that each node is assigned a unique peak, and that no two nodes are assigned the same peak. We reach

$$\begin{aligned} {\mathbb{P}}({\mathcal{D}}| {\mathcal{T}})&= {} {\mathbb{P}}(\text{all peaks in }{\mathcal{D}}\text{ are noise})\\&\quad \prod \limits _{v \in V} \frac{{\mathbb{P}}(m(v) | v)}{{\mathbb{P}}(m(v) \text{is noise})} \end{aligned}$$

for appropriate ${\mathbb{P}}(m(v) | v)$. Again, for fixed data ${\mathcal{D}}$, the probability of all peaks being noise simultaneously is a constant, and can be ignored in the optimization of ${\mathbb{P}}({\mathcal{T}}| {\mathcal{D}})$.

We will now show how to compute the probability of signal peaks and noise peaks. Currently, there exists no general model for the intensity of signal peak in small compound MS. Here, the problem is even harder, as we do not know the fragment’s molecular *structure* but only its molecular formula. Similarly, there exists no sensible model for the mass of noise peaks. To this end, we will use only the peak mass to assess the probability of signal peaks; and only peak intensity to assess the probability of noise peaks. The intensity of peak *m* is *I*(*m*);  for brevity we write $I(v) := I(m(v))$.

#### Probability of signal peaks

It has been frequently observed that relative mass deviations are roughly normally-distributed [57, 58]. We found this to be the case for our datasets, see “Statistics and fitting the model” section. We assume that the instrument is decently calibrated, so that no mass bias can be observed. Let $MA$ be the mass accuracy parameter used to build the fragmentation graph. If we assume that 95.5 % of the normally-distributed masses fall within this range, then the standard deviation is $\sigma _{\text{m}}:= \frac{1}{2} MA ;$ if we assume that 99.7 % of the masses fall within this range, then $\sigma _{\text{m}}:= \frac{1}{3} MA .$ Now, relative mass errors are distributed according to ${\mathcal{N}}(0,\sigma _{\text{m}}).$ We ignore the fact that no mass errors above some threshold can be observed (truncated normal distribution) as this has a negligible effect on our computations. The probability to observe a peak with mass *m*(*v*) for node/fragment *v* can be estimated as

8 $$\begin{aligned} {\mathbb{P}}(m(v) | v)&= {} {\mathbb{P}}\left( \left|{\mathcal{N}}(0,\sigma _{\text{m}})\right| \ge \tfrac{\left|m(v) - \mu (v)\right|}{\mu (v)} \right) \nonumber \\&= {} 2 \cdot \int _{\frac{\left|m(v) - \mu (v)\right|}{\mu (v)}}^{\infty } \frac{1}{\sigma _{\text{m}}\sqrt{2\pi }} e^{ -\frac{1}{2} \left( \frac{x}{\sigma _{\text{m}}}\right) ^2 } dx\nonumber \\&= {} {{\mathrm{erf}}}\left( \tfrac{\left|m(v) - \mu (v)\right|}{\sigma _{\text{m}}\sqrt{2} \mu (v)} \right) . \end{aligned}$$

This is the two-sided probability that a mass deviation larger than the observed relative mass deviation of peak *m*(*v*) will occur by chance. Here, “${{\mathrm{erf}}}$” denotes the error function.

#### Probability of noise peaks

As we have no model for the intensity of fragment peaks, *I*(*v*) cannot be used for estimating the probability of fragment peaks. Similarly, we have no model for noise peak masses. But we can estimate the probability that a certain peak is noise, by observing that noise with high intensity are much rarer than noise peaks with small intensity.

Böcker and Rasche [34] proposed to directly use the peak intensity in the score calculation. Later, Rasche et al. [41, Suppl. Material] pointed out that this can be statistically justified by assuming that noise peak intensities are exponentially distributed. To this end, we analyze the intensity distribution of noise peaks, see “Statistics and fitting the model” section. We observe that with increasing intensity, the probability to observe a noise peak of this intensity drops rapidly in the beginning, but stays significantly above zero even for large intensities. This is an example of a long-tailed distribution, and we use the Pareto distribution as a classical example of a long-tailed distribution. This distribution offers the additional advantage that a minimum peak intensity threshold, which is naturally applied in peak picking, can be directly integrated into the model.

Let $x_{\text{i}}$ be the peak intensity threshold used for peak picking. Then, the probability density function of the Pareto distribution is $\alpha _{\text{i}}x_{\text{i}}^{\alpha _{\text{i}}}{/}x^{\alpha _{\text{i}}+1}$ for mass *x*. See “Statistics and fitting the model” section for fitting hyperparameters $\alpha _{\text{i}},x_{\text{i}}$. Then, the probability of observing a noise peak *m* with intensity *I* or higher, is

9 $${\mathbb{P}}(m\text{ is noise}) = \frac{\alpha _{\text{i}}x_{\text{i}}^{\alpha _{\text{i}}}}{I^{\alpha _{\text{i}}+1}}.$$

We found that the Pareto distribution agrees well with the experimental data, see “Statistics and fitting the model” section.

### Posterior probability of the tree

From the above we infer that

10 $$\begin{aligned}{\mathbb{P}}(T) &\cdot {\mathbb{P}}({\mathcal{T}}| {\mathcal{D}}) \varpropto P_{\mathrm{root}}(r) \cdot \prod _{e \in E} (P_\mathrm{edge}(e) \cdot P_{\text{tree-size}})\nonumber \\& \cdot \prod _{v \in V} \left( {{\mathrm{erf}}}\left( \tfrac{\left|m(v) - \mu (v)\right|}{\sigma _{\text{m}}\sqrt{2} \mu (v)} \right)\left/\tfrac{\alpha _{\text{i}}x_{\text{i}}^{\alpha _{\text{i}}}}{I(v)^{\alpha _{\text{i}}+1}} \right)\right.\end{aligned}$$

for FT ${\mathcal{T}}= (V,E)$ with root $r \in V$. The probability that all peaks in the spectrum are noise, is independent of the actual tree ${\mathcal{T}}$ and, hence, can be disregarded. We define

11 $$\begin{aligned} {\mathcal{L}}({\mathcal{T}})&:= {} \log P_{\mathrm{root}}(r) + \sum _{e \in E} \log \left( P_{\mathrm{edge}}(e) \cdot P_{\text{tree-size}}\right) \nonumber \\&\quad + \sum _{v \in V} \left( \log {{\mathrm{erf}}}\left( \tfrac{\left|m(v) - \mu (v)\right|}{\sigma _{\text{m}}\sqrt{2} \mu (v)} \right) - \log \tfrac{\alpha _{\text{i}}x_{\text{i}}^{\alpha _{\text{i}}}}{I(v)^{\alpha _{\text{i}}+1}} \right) \end{aligned}$$

then $\log ({\mathbb{P}}(T) \cdot {\mathbb{P}}({\mathcal{T}}| {\mathcal{D}})) = {\mathcal{L}}({\mathcal{T}}) + c$ for some constant $c \in {\mathbb{R}}$. To this end, the posterior probability of tree ${\mathcal{T}}$ is maximum if and only if ${\mathcal{L}}({\mathcal{T}})$ is maximum.

Given a fragmentation spectrum ${\mathcal{D}}$ we proceed as follows: first, for each peak $m \in {\mathcal{D}}$ we search for all molecular formulas *v* that are within the specified mass accuracy $MA,$

$$\mu (v) \in \left[ m - \delta , m + \delta \right] .$$

where $\delta = MA \cdot \max \{m,m_{ MA }\}.$ In case two of these intervals overlap, we shrink them accordingly. We use these molecular formulas *v* as the nodes of the fragmentation graph, each colored with the corresponding mass *m*, and set $m(v) = m$. We introduce an edge (*u*, *v*) for each pair $u \ge v$. For each edge (*u*, *v*) we set its edge weight

12 $$\begin{aligned} w(u,v)&:= {} \log P_{\mathrm{edge}}(u,v) + \log P_{\text{tree-size}}\nonumber \\&\quad + \log {{\mathrm{erf}}}\left( \tfrac{\left|m(v) - \mu (v)\right|}{\sigma _{\text{m}}\sqrt{2} \mu (v)} \right) - \log \tfrac{\alpha _{\text{i}}x_{\text{i}}^{\alpha _{\text{i}}}}{I(v)^{\alpha _{\text{i}}+1}}. \end{aligned}$$

We also introduce a super-root $sr$ which is connected to all nodes corresponding to the parent mass *M*. These $v \in V$ with $m(v) = M$ are the potential roots of the FT, and for each we set

13 $$\begin{aligned}w( sr ,v) &:= \log P_{\mathrm{root}}(v)\\ &\quad + \log {{\mathrm{erf}}}\left( \tfrac{\left|m(v) - \mu (v)\right|}{\sigma _{\text{m}}\sqrt{2} \mu (v)} \right) - \log \tfrac{\alpha _{\text{i}}x_{\text{i}}^{\alpha _{\text{i}}}}{I(v)^{\alpha _{\text{i}}+1}}.\end{aligned}$$

With these edge weights, ordering colorful subtrees with respect to their weight, is equivalent to ordering the corresponding FTs by posterior probability.

### Hypothesis-driven recalibration

To improve the quality of FTs, and to increase the chance that the FT with the correct molecular formula root will receive the highest score, we use a hypothesis-driven recalibration [47]. We are given one fragmentation spectrum at a time. For each candidate molecular formula explaining the root, we compute a FT, and then use the theoretical masses of all nodes in the FT as references to recalibrate the sample spectrum. Some of the molecular formulas assigned to peaks may be wrong, even for the correct candidate molecular formula. To this end, we use recalibration methods which are robust to outliers, and automatically discard such wrong assignments when computing the recalibration.

Recalibration is carried out using an affine mass correction [47] $f(x) := ax + b.$ Let $(x_i,y_i)$ be the pairs of potentially matching masses: $x_i$ is a mass in the measured spectrum, and $y_i$ is a mass in the reference spectrum simulated using the FT. Note that for any measured (reference) mass there can be multiple elements with different reference (measured) masses. We use the Theil-Sen estimator [59, 60] to find the slope *a* of *f* as the median of the slopes $(y_j - y_i){/}(x_j - x_i)$ determined by all pairs of sample points with distinct x-coordinates. Next, we set *b* to be the median of the values $y_i - mx_i$. We recalibrate the measured spectrum by applying *f* to all masses.

We then compute the optimal FT for the recalibrated sample spectrum and the candidate molecular formula, and use this score to evaluate which root molecular formula best explains the data. Then, the recalibration is *discarded*, returning to the original measured sample spectrum, and the next root molecular formula is processed.

We note that our hypothesis-driven recalibration (HDR) is fundamentally different from, say, the recalibration proposed in [49]: using HDR, each spectrum is recalibrated individually, using each peak’s best theoretical explanation as anchors for the mass correction. In this way, we do not require a homogeneous dataset of mass spectra to start the recalibration process.

### Statistics and fitting the model

We now describe how to estimate the (hyper)parameters for priors and the likelihood estimation. Parameters for mass error and peak intensity can be chosen individually for each dataset, and are required for computing the likelihood of the data. In contrast, hyperparameters are only estimated once for the SIRIUS 3 method, and are not retrained for a new dataset; they are required for computing the prior probability of a FT.

#### Mass error and noise peak intensity

Mass accuracy parameters $\sigma _{\text{m}}, MA , m_{ MA }$ and noise intensity parameters $x_{\text{i}}, \alpha _{\text{i}}$ can be determined individually for each dataset. For example, these parameters can be chosen by manual inspection of the data. SIRIUS 3 can achieve good performance even if parameters deviate significantly from the experimental truth, but better estimates will usually further improve the method’s power (see Table 1).

For both datasets in our evaluation, we estimate $MA = 10$ ppm, $m_{ MA } = 200$ Da, and $\sigma _{\text{m}}= 10$ by manual inspection of the data. To avoid overestimating the method’s power as well as overfitting, we do not train these parameters.

**Fig. 11:**
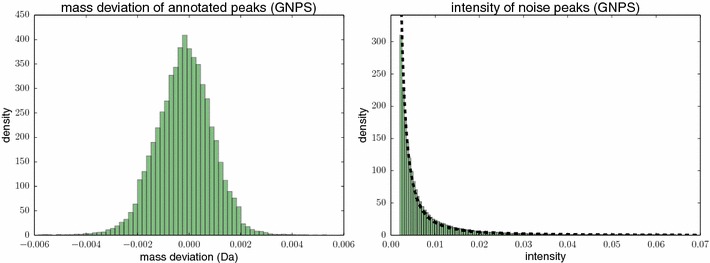
*Left* normalized histogram of the mass error distribution, for the GNPS dataset. *Right* normalized histogram of the noise peak intensity distribution and fitted Pareto distribution (*dashed line*), for the GNPS dataset

Is our assumption correct that mass errors are normally distributed? To verify this claim, we have to know the true (theoretical) mass of the fragments that resulted in some peak in the spectrum. To estimate the true mass, we use FTs computed for the correct root molecular formula, after setting all hyperparameters as described below. We compare the theoretical mass of each FT node with the observed mass of the peak. For both datasets, we observe that mass errors roughly follow a normal distribution (see Fig. 11). We find that the standard deviation of this distribution is somewhat smaller than the manual chosen parameter $\sigma _{\text{m}}$.

We have to determine the noise intensity parameters individually for each of the two datasets, because spectra in the Agilent dataset only provide relative intensities, whereas GNPS spectra provide absolute intensities.

We use the peak intensity threshold $x_{\text{i}}= 0.002$ for GNPS and $x_{\text{i}}= 0.005$ for Agilent as the first parameter of the noise intensity Pareto distribution. Parameter $\alpha _{\text{i}}$ can be estimated from the data using peaks that have no decomposition as a sub-formula of the known molecular formula of the compound, or masses larger than the mass of the precursor peak: these peaks are generally noise peaks. (In case no reference compounds are known in the dataset, we can instead choose those peaks that have no decomposition whatsoever.) We plot relative noise peak intensities in Fig. 11 (right). In both datasets, we observe a rapid decay of noise peaks with increasing intensity. We estimate $\alpha _{\text{i}}= 0.34$ for GNPS and $\alpha _{\text{i}}= 0.5$ for Agilent, see Fig. 11. The larger $\alpha _{\text{i}}$ for Agilent is probably an artifact of intensity normalization: if the most intense peak in a spectrum has a low intensity, which happens frequently in high-energy spectra, all other peaks (including noise peaks) have comparatively large relative intensities.

#### Iterative estimation of hyperparameters

Recall that hyperparameters are estimated only once for the SIRIUS 3 method, and are not retrained for a new dataset. We estimate hyperparameters from FTs that we have previously computed by SIRIUS 3. Clearly, for a FT to carry useful information, it has to be the FT that carries the true molecular formula of the compound as its root. For the rest of this chapter, FTs will always be computed for the true molecular formula. We estimate the hyperparameters only once using all FTs from both datasets, instead of estimating hyperparameters for each dataset individually.

We re-estimate hyperparameters in an iterative procedure, consisting of *rounds*: for the first round, we manually set parameters $\mu _{\text{ls}}= 4$ and $\sigma _{\text{ls}}= 0.5$ for the loss mass distribution, which are estimated from FTs computed using SIRIUS^2^-ILP [34, 41, 42]. We also use the manually derived list of common losses [42] with scores that compensate for 75 % of the penalty through the loss mass distribution. (Both of these estimates differ strongly from those that result from our iterative estimation procedure, indicating its robustness.) In this first round, the list of common fragments is empty, and the tree size prior is set to $P_{\text{tree-size}} = e^5 = 148.41$ to counter the effect of the other priors. We then compute a first round of FTs with these priors.

Using these FTs, we estimate the loss mass prior, the common losses, the common fragments, and the tree size prior as described below. We then iterate: using these new priors, we again compute FTs, and proceed by recomputing the hyperparameters. We repeat this for *ten rounds*.

#### Estimating the loss mass distribution and common losses

We now consider the set of all losses that have been observed in at least one tree, together with their number of appearances (frequency). But instead of purely counting losses, we want to give more weight to losses that correspond to intense peaks. To this end, any loss receives weight corresponding to the maximum peak intensity of the two peaks that are responsible for this loss.

Loss mass distribution and the list of common losses are jointly determined in an inner loop: the loss mass distribution dictates what losses we regard as being “more common than expected”. But these common losses, in turn, have to be made “uncommon” for determining the loss mass distribution. We proceed in six runs.

Let $l_1,\ldots ,l_N$ be the observed losses, $x_1,\ldots ,x_N$ the loss masses, and $w_1,\ldots ,w_N$ the corresponding weights reflecting peak intensities. We may assume that all losses $l_k$ are pairwise different, summing up weights. Let *w*(*l*) be the total weight of some loss $l := u-v.$ Further, set $w^{\prime }_k \leftarrow w_k$ for all $k = 1,\ldots ,N$; these will be the weights that are updated in each run. Maximum likelihood estimates of $\mu _{\text{ls}}, \sigma _{\text{ls}}$ are

14 $${\widehat{\mu }} = \frac{\sum \nolimits _k w^{\prime }_k \ln x_k}{W}, \quad {\widehat{\sigma }}^2 = \frac{\sum \nolimits _k w^{\prime }_k \left( \ln x_k - \widehat{\mu }\right) ^2}{W}$$

where $W := \sum \nolimits _k w^{\prime }_k$ is the *total weight* of all observed losses. We set $\mu _{\text{ls}}= \widehat{\mu }$ and $\sigma _{\text{ls}}= \widehat{\sigma }$ for (6).

Certain losses appear significantly more often than we would expect from the loss mass distribution. To this end, we use the following two rules to decide whether some loss $l := u-v$ is termed “common”:

1. The observed sum of weights for this loss is at least 1.3-fold of what we would expect from (6).
2. Large losses will be very rare and, using only the above rule, all of them would be regarded as “common”. To this end, we also demand that the frequency have to be at least five above the expected value from (6).

Common losses are outliers, in the sense that their frequency is far higher than we would expect for a loss of this mass. To this end, we now correct their weight in a straightforward manner: for each identified common loss $l_k$, we set its weight to exactly the value we would expect from the loss mass prior, namely $w^{\prime }_k \leftarrow P_{\text{ loss-mass}}(\mu (l)) \cdot W.$

After the *final round* of fitting the hyperparameters, we reach $\mu _{\text{ls}}= 4.02$ and $\sigma _{\text{ls}}= 0.31.$ The mode of the log normal distribution is mass $e^{\mu _{\text{ls}}} = 55.84\,\hbox {Da}$. For each loss *l* in the identified list of common losses, we set:

$$P_{\text{loss-comm}}(l) := \frac{w(l)}{P_{\text{loss-mass}} (\mu (l)) \cdot W}$$

Losses H and $\hbox {H}_{2}$ are special cases, as they have very low prior probabilities due to the loss mass prior being a log normal distribution. We set the common loss prior for both losses such that the losses are neither penalized nor favored: in detail, the product of the priors is equal to the geometric mean of the product for all other losses.

**Fig. 12:**
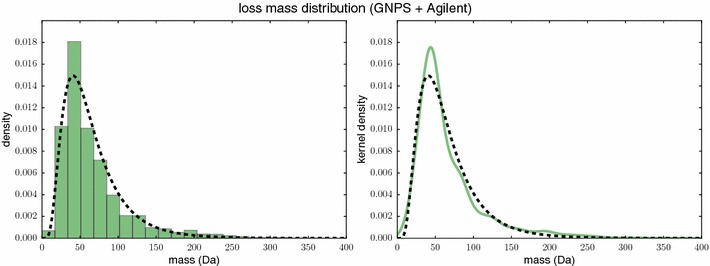
Loss mass distribution, after the final round of parameter estimation. Frequencies of the losses are weighted by the intensity of their peaks. The frequency of the identified common losses have been decreased to the value of the log-normal distribution. *Left* normalized histogram for bin width 17 Da (*green*). *Right* kernel density estimation (*green*). Maximum likelihood estimate of the log-normal distribution drawn in both plots (*black*, *dashed*)

See Fig. 12 for the agreement between the observed distribution of loss masses (corrected for common losses as indicated above), and the fitted log-normal distribution. See Table 3 for the list of identified common losses. We find that the resulting list of common losses shows high agreement with the expert-curated lists from [41, 42].

#### Common fragments

After loss mass distribution and common losses are determined for the current round, we determine a list of common fragments that show up significantly more often then what we would expect by chance.

For each fragment, we compute its weight as the sum of peak intensities of the corresponding peaks. Then, we compute a frequency of each fragment, dividing its weights by the total weight of all fragments. Unlike losses, the diversity of fragments is very high (we observed 13,537 different fragments in our datasets, most of them occurring only one time). To avoid overfitting, we use only the 40 most common fragments and set the common fragment prior to their weight divided by the weight of the 80th most common fragment (39). Both numbers are chosen ad hoc. All other fragments get a flat prior of 1. See Table 4 for the resulting common fragments.

#### Tree size prior

Finally, we determine tree size priors $P_{\text{tree-norm}}$ and $P_{\text{tree-bonus}}$: we choose $P_{\text{tree-norm}}$ as the inverse of the geometric mean of the priors that any edge in any FT receives. The more interesting prior is $P_{\text{tree-bonus}}$ that can be used to control the size of the trees. We want to ensure that a high percentage of peaks in the fragmentation spectra are explained by our FTs. For the first round we set $P_{\text{tree-bonus}} \leftarrow 1$. In the following rounds we decrease $P_{\text{tree-bonus}}$ by dividing it with $e^{0.25}.$ We then re-compute FTs with the new priors of the current round. To decide whether we have explained “enough” peaks, we use the following criterion: we compute the sum of intensities of all peaks that are explained by the FTs. We also compute the sum of intensities of all peaks that *could be* explained by a theoretical fragment, that is, $\left|m-m^{\prime }\right| \le MA \cdot \max \{m,m_{ MA }\}$ for peak mass *m* and molecular formula mass $m^{\prime }$. If the ratio of explained intensities versus intensities that could be explained, drops below 85 % then we increase $P_{\text{tree-bonus}}$ by multiplying it with $e^{0.5},$ and re-start the computation of FTs. As soon as this ratio is above 85 %, we keep the FTs and proceed to the next round.

After the final found, we reach tree size priors $P_{\text{tree-norm}} = e^{1.46}$ and $P_{\text{tree-bonus}} = e^{-0.5}.$

## Footnotes

1 Stravs et al. [49] wrongly conjectured that SIRIUS^2^ used additional chemical rules to filter molecular formulas, but the inability of SIRIUS^2^ to process certain instances is apparently simply a bug.
